# Mesenchymal Stromal Cell Secretome and Its Key Bioactive Metabolites Induce Long‐Term Neuroprotection After Traumatic Brain Injury in Mice

**DOI:** 10.1002/advs.202415508

**Published:** 2025-06-19

**Authors:** Francesca Pischiutta, Francesca Tribuzio, Marta Magatti, Giulia De Simone, Federico Moro, Giovanni Nattino, Fabiola Signorini, Luther Loose, Enrico Caruso, Costanza Bertani, Edoardo Mazzone, Rosaria Pascente, Edoardo Micotti, Antonietta Rosa Silini, Fabrizio Ortolano, Maria Chiara Trolese, Marco Bolis, Luca Guarrera, Martina Bruna Violatto, Paolo Bigini, Cristina Banfi, Roberta Pastorelli, Ornella Parolini, Laura Brunelli, Elisa R Zanier

**Affiliations:** ^1^ Laboratory of Traumatic Brain Injury and Neuroprotection Department of Acute Brain and Cardiovascular Injury Istituto di Ricerche Farmacologiche Mario Negri IRCCS Milan Italy; ^2^ Centro di Ricerca E. Menni Fondazione Poliambulanza – Istituto Ospedaliero Brescia Italy; ^3^ Laboratory of Protein and Metabolites in Translational Research Department of Environmental Health Science Istituto di Ricerche Farmacologiche Mario Negri IRCCS Milan Italy; ^4^ Laboratory of Clinical Epidemiology Department of Medical Epidemiology Istituto di Ricerche Farmacologiche Mario Negri IRCCS Ranica Bergamo Italy; ^5^ Laboratory of Biology of Neurodegenerative Disorders Department of Neuroscience Istituto di Ricerche Farmacologiche Mario Negri IRCCS Milan Italy; ^6^ Neuroscience Intensive Care Unit Department of Anesthesia and Critical Care Fondazione IRCCS Ca' Granda Ospedale Maggiore Policlinico Milan Italy; ^7^ Computational Oncology Unit Experimental Oncology Department Istituto di Ricerche Farmacologiche Mario Negri IRCCS Milan Italy; ^8^ Laboratory of Nanobiology Department of Molecular Biochemistry and Pharmacology Istituto di Ricerche Farmacologiche Mario Negri IRCCS Milan Italy; ^9^ Unit of Functional Proteomics Metabolomics, and Network analysis Centro Cardiologico Monzino IRCCS Milan Italy; ^10^ Department of Life Science and Public Health Università Cattolica del Sacro Cuore Rome Italy; ^11^ Fondazione IRCCS Casa Sollievo della Sofferenza San Giovanni Rotondo Foggia Italy

**Keywords:** immunomodulation, mesenchymal stromal cells, metabolomics, neuroprotection, secretome, traumatic brain injury

## Abstract

The severe and long‐term consequences of traumatic brain injury (TBI) highlight the urgent need for effective neuroprotective therapies. Mesenchymal stromal cells (MSCs) show promise in TBI treatment through their secretome (conditioned media, CM). A low‐molecular‐weight (<700 Da) CM fraction with neuroprotective effects comparable to total CM after acute brain injury in vitro is previously identified. Here, it is aimed at identifying key bioactive factors, reconstituting them into a synthetic cocktail (SYNT), and evaluating its efficacy in TBI models. Metabolomic profiling identified three prostaglandins and kynurenine, which are used to create SYNT. The SYNT formulation reduced cell death, neuronal damage, and induced protective gene expression changes associated with neuronal protection and microglia modulation toward beneficial phenotype after TBI in vitro. In vivo, SYNT conferred similar long‐term functional benefits as CM, improving sensorimotor function up to 6 months and memory preservation at 4 months compared to saline‐treated animals, though only CM reduced contusion volume at 5 months. Both treatments modulated neuroinflammation, evidenced by reduced microglial activation and astrogliosis in the pericontusional tissue at 6 months. These findings demonstrate the neuroprotective effects of MSC‐secretome treatment in TBI and highlight prostaglandins and kynurenine as key mediators of this response. The findings lay the groundwork for developing a standardized, cell‐free therapeutic strategy for TBI based on MSC derivatives.

## Graphical abstract

Pischiutta F and coworkers identify prostaglandins and kynurenine as key mediators of the mesenchymal stromal cells‐derived secretome's neuroprotective effects. A synthetic cocktail composed of these factors promotes long‐term functional recovery, reduces brain structural damage, and modulates neuroinflammation after traumatic brain injury, supporting the development of standardized, cell‐free therapeutic strategies.

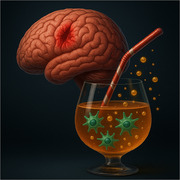

## 1 Introduction

Traumatic brain injury (TBI) is a major global health challenge, contributing to significant morbidity and mortality worldwide.^[^ ^1^ ^]^ The complex pathology of TBI necessitates multitarget therapeutic strategies^[^ ^2^ ^]^ to simultaneously mitigate harmful pathways and promote the protective and regenerative responses. Mesenchymal stromal cells (MSCs) have emerged as a promising therapeutic option due to their unique biological properties and ability to modulate immune responses and support tissue repair.^[^ ^3^ ^]^ MSCs reduce neutrophil infiltration, inhibit T‐cell proliferation, and promote a pro‐regenerative macrophage profile.^[^ ^4^, ^5^ ^]^ They also secrete growth factors and cytokines that enhance angiogenesis, neurogenesis, and reduce inflammation.^[^ ^3^ ^]^ Preclinical studies consistently demonstrate MSC efficacy in TBI models across various MSC sources, treatment protocols, and TBI experimental models.^[^ ^6^ ^]^ Clinically, MSCs have shown high safety profile in many pathologic conditions,^[^ ^7^, ^8^, ^9^ ^]^ even in critically ill patients.^[^ ^10^ ^]^ Building on this foundation, we are now conducting the MATRIx phase II trial to evaluate the safety and biological effects of allogeneic MSCs in severe TBI patients.^[^ ^11^ ^]^

There is evidence that MSC‐released factors, collectively known as the secretome, drive these therapeutic effects. We previously demonstrated that human amniotic membrane MSCs (hAMSCs) protect the traumatized brain independently from direct cell‐brain interaction.^[^ ^12^ ^]^ In a recent meta‐analysis on preclinical models of TBI, we found that MSC derivatives produced therapeutic effects comparable to, or even exceeding those of the cells themselves.^[^ ^6^ ^]^ This suggests the potential for a cell‐free approach centered on the MSC secretome. Moreover, in an in vitro model of acute brain injury, we observed the protective effects of MSC‐secretome.^[^ ^12^ ^]^ Seeking to isolate the most active components, we identified a secretome subfraction composed of molecules smaller than 700 Daltons, which showed significant neuroprotective activity^[^ ^12^ ^]^ supporting the rationale for the development of a cell‐free strategy leveraging MSC‐derived factors.

By characterizing the composition of the secretome, we open the possibility of creating a synthetic cocktail of bioactive molecules that offers several advantages over traditional MSC‐based therapies. This approach is potentially more scalable, reducing donor variability and the need for expensive, good manufacturing practice (GMP)‐compliant facilities. Moreover, it enables the production of a readily available, cost‐effective treatment that can be administered widely and without the logistical challenges associated with live cell therapies. However, identifying the bioactive components responsible for neuroprotection remains a key challenge. In this study, we aimed to: 1) evaluate the neuroprotective effects of MSC‐secretome and its <700 Da subfraction in TBI mice leveraging previous results;^[^ ^12^ ^]^ 2) characterize the metabolomic profile of the protective fraction; and 3) reconstruct a synthetic cocktail (SYNT) for testing in in vitro and in vivo TBI models.

## 2 Results

### 2.1 Conditioned Media from hAMSC is Protective After TBI

**Figure 1:**
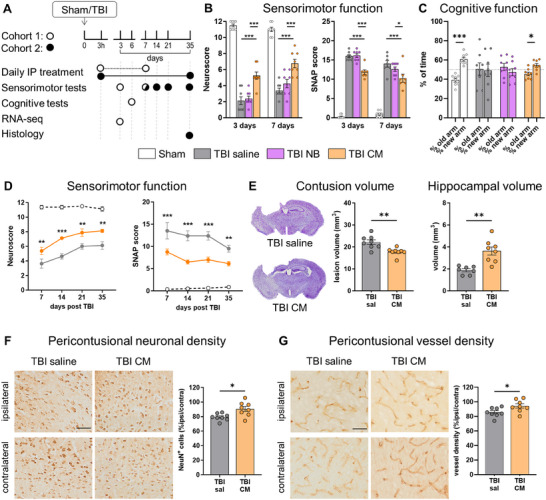
CM efficacy in TBI mice. A) Schematic representation of the experimental design of Cohort 1 (white), aimed at defining the specificity of CM protective effects, and Cohort 2 (black), aimed at investigating CM efficacy up to 1‐month post‐injury. B) Sensorimotor assessment at 3‐ and 7‐days post TBI by Neuroscore and Simple Neuroassessment of Asymmetric imPairment (SNAP) of Cohort 1. C) Cognitive assessment at 6 days post TBI by Y maze test of Cohort 1. D) Sensorimotor assessment up to 1‐month post TBI by Neuroscore and SNAP tests of Cohort 2. E) Representative Nissl‐stained coronal sections and quantification of contusion volume and hippocampal volume 1‐month post‐injury (Cohort 2). F) Microphotographs showing NeuN staining in the ipsilateral (pericontusional) and contralateral cortical tissue of TBI saline and TBI CM mice, 1‐month post‐injury (Cohort 2), and relative quantification. G) Microphotographs showing CD31 staining in the ipsilateral (pericontusional) and contralateral cortical tissue of TBI saline and TBI CM mice, 1‐month post‐injury (Cohort 2), and relative quantification. Data are presented as mean ± SEM; n = 7,8 mice/group. B, D) Two‐way ANOVA for repeated measurement, followed by Tukey post‐test. C, E, F, G) t‐test. *p < 0.05, **p < 0.01, ***p < 0.001. Bar = 50 µm.

We initially evaluated the efficacy and specificity of hAMSC‐secretome collected in the conditioned media (CM) after TBI using the experimental design in **Figure** **1**A (Cohort 1). TBI mice were treated daily with saline, neurobasal (NB), or CM (150 µL intraperitoneally, IP), starting from 3 h post‐injury. NB treatment served as control to exclude the potential influence of nutrients present in the culture medium. TBI mice receiving either saline or NB showed comparable behavioral impairments at neuroscore and Simple Neuroassessment of Asymmetric imPairment (SNAP) tests at both 3‐ and 7‐days post‐injury. However, mice treated with CM exhibited significant early sensorimotor improvements at both tests (Figure 1B). Likewise, cognitive assessment at Y‐maze test revealed no memory retention in TBI saline and NB groups; whereas TBI CM‐treated mice showed cognitive improvements indicated by more time spent in the new versus old arm (Figure 1C). No differences were observed among groups in locomotor activity or anxiety‐like behavior (Supplementary Figure S1A, Supporting Information). These data indicate that CM promotes recovery of sensorimotor and mnestic functions through specific molecules released by hAMSC.

To investigate microenvironmental changes induced by CM acutely after injury, we performed RNA sequencing (RNA‐seq) 3 days post‐TBI of the pericontusional cortex of TBI saline and TBI CM mice. The GSEA revealed a CM‐induced upregulation of pathways related to collagen degradation (Reactome Collagen Degradation) and ECM degradation (Reactome Degradation of the Extracellular Matrix), highlighting active ECM modulation in response to CM treatment (Supplementary Figure S2, Supporting Information).

We further assessed CM efficacy over a longer period (up to 1 month, Figure 1A, Cohort 2). CM‐treated mice showed sustained improvements in sensorimotor function (Figure 1D) and a significant reduction of contusion volume and hippocampal damage (Figure 1E). Histopathological analysis revealed CM‐induced neuronal protection (Figure 1F) and vessel rescue (Figure 1G) in the perilesional cortex. These results demonstrate the protective effects of hAMSC‐derived CM in vivo after experimental TBI, supporting further analyses of its composition.

To explore whether direct brain delivery could enhance therapeutic efficacy, we administered CM intracerebroventricularly (ICV) at 3 h post injury. While ICV‐treated mice showed improved sensorimotor function, effect size was lower than that observed after repeated IP administration, and no significant reduction in contusion volume was observed (Supplementary Figure S3, Supporting Information).

### 2.2 CM Fraction <700 Da is Protective After TBI: Metabolomic Analysis

**Figure 2:**
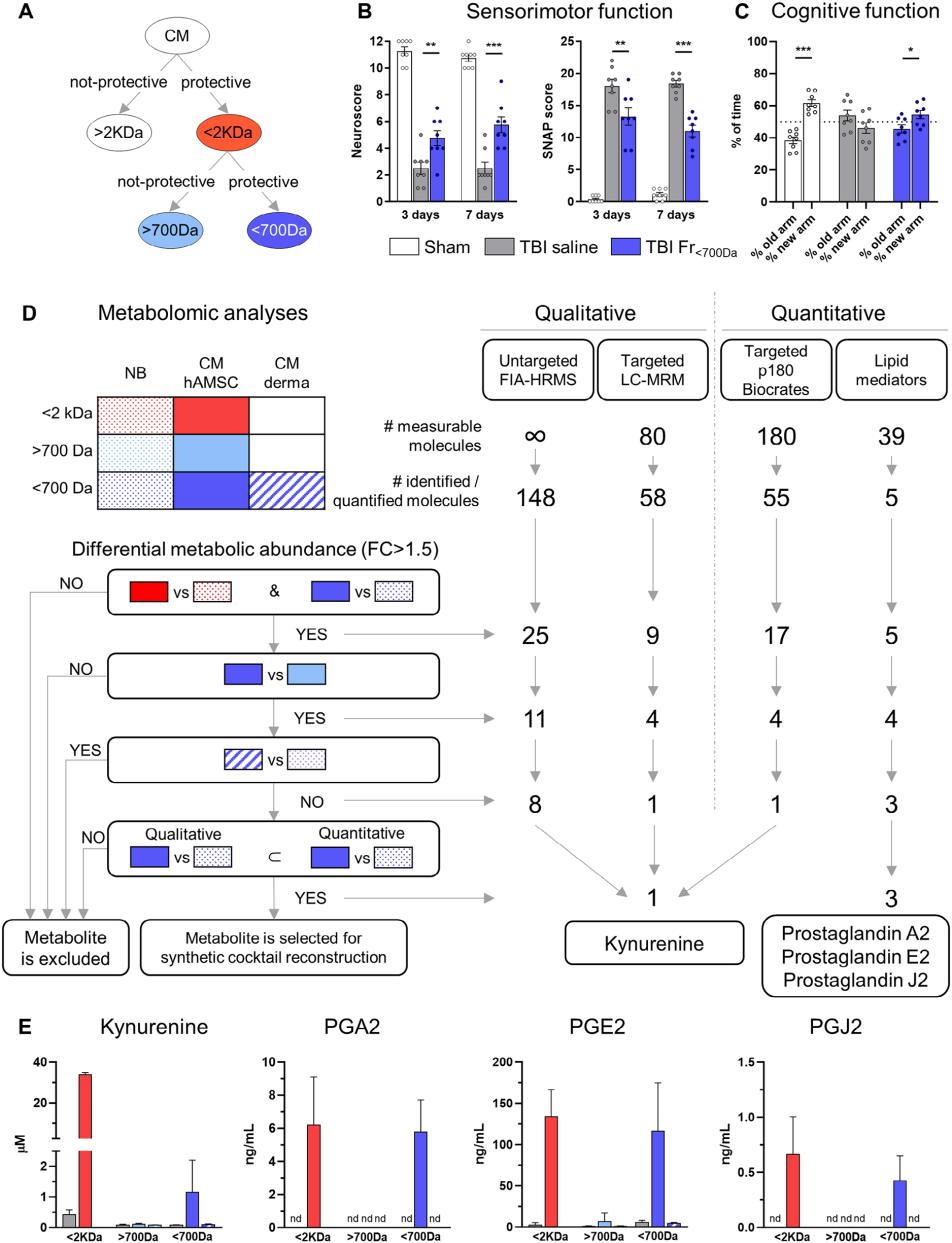
Efficacy of CM fraction <700 Da in TBI mice and omics analyses. A) Schematic representation of the CM fractions previously found to be protective or non‐protective (>700 Da or CM derma) in an in vitro model of acute brain injury. B) Sensorimotor assessment at 3‐ and 7‐days post TBI by Neuroscore and Simple Neuroassessment of Asymmetric imPairment (SNAP). C) Cognitive assessment at 6 days post TBI by Y maze test. Data are presented as mean ± SEM; n = 8. B) Two‐way ANOVA for repeated measurement, followed by Tukey post‐test. C) t‐test. *p < 0.05, **p < 0.01, ***p < 0.001. D) Schematic of the color codes used for fractions <2 KDa, >700 Da, and <700 Da obtained from NB, CM‐hAMSC, and CM‐derma. Fractions were analyzed by 4 metabolomic analyses: 2 qualitative (FIA‐HRMS and LC‐MRM) and 2 quantitative (p180 Biocrates kit, Lipid mediators). The decision tree illustrates the number of measurable molecules for each technique, the number of identified molecules, and the gerarchic process used to select metabolites for synthetic cocktail reconstruction. FC = fold change of abundance. E) Graphs represent the concentration of the selected metabolites.

Our previous work identified a CM fraction containing molecules smaller than 700 Da (Fr <700 Da) with neuroprotective effects after acute brain injury in vitro (CM fractionation with sequential size‐exclusion ‐cut‐off 2 kDa‐ and gel‐filtration chromatography is illustrated in **Figure** **2**A).^[^ ^12^ ^]^ To determine if this fraction <700 Da retained its efficacy in vivo, we tested its effects in TBI mice (Cohort 3) using the same protocol as in Cohort 1 (experimental design in Figure 1A). Treatment with Fr<700 Da induced early significant improvements in sensorimotor function (Figure 2B) and cognitive performance (Figure 2C) compared to saline, while no differences were found in terms of locomotor activity or anxiety‐like behavior (Supplementary Figure S1B, Supporting Information). These data demonstrate that protective mediators are contained in the CM fraction <700 Da. Using metabolomics analyses (qualitative: Flow Injection Analysis High resolution mass spectrometry, FIA‐HRMS and Liquid Chromatography‐multiple reaction monitoring  LC‐MRM; and quantitative: p180 Kit Biocrates, Lipid mediators), we compared the <700 Da fraction from CM obtained from 3 different batches, with the corresponding fractions from NB and fibroblast‐derived CM (CM‐derma), which lack protective effects.^[^ ^12^ ^]^ Metabolites were selected based on differential abundance (fold change >1.5) following a decision tree approach (Figure 2D). This analysis identified four metabolites selectively enriched in CM fraction <700 Da: kynurenine, prostaglandin (PG) A2, PGE2, and PGJ2 (Figure 2E).

### 2.3 The Synthetic Cocktail is Protective After TBI In Vitro

**Figure 3:**
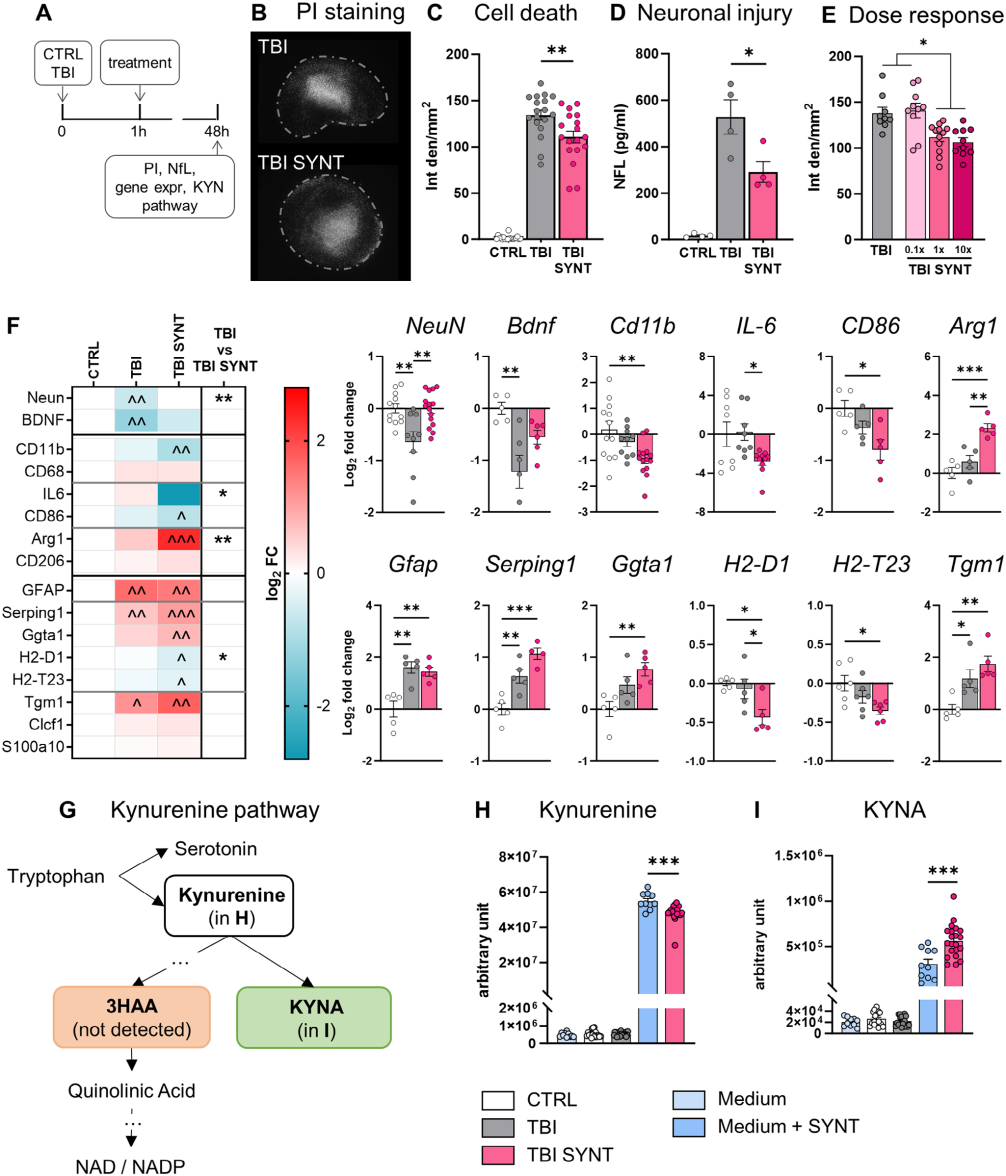
Efficacy of synthetic cocktail after TBI in vitro. A) Schematic representation of the experimental design used for the assessment of synthetic cocktail (SYNT) efficacy after TBI in vitro. B) Representative images showing propidium iodide (PI) incorporation 48 h after injury in slices subjected to TBI or TBI + synthetic cocktail. C) Quantification of PI incorporation 48 h after injury. D) Quantification of NfL released in the culture medium at 48 h post‐injury, as an index of neuronal injury. E) Dose response effects using SYNT at concentration 0.1x, 1x, 10x. F) Gene expression analysis of brain slices 48 h post‐injury represented as heat map (left) or as graphs (only for genes with statistical significance, right). Expression of the neuronal marker *NeuN*, the trophic factor brain‐derived neurotrophic factor (*BDNF*), the microglial (*CD11b, CD68*) with pro‐inflammatory M1‐like (*IL‐6, CD86*) and M2‐like (*Arg1*, CD206) related markers and the astrocytic (*GFAP*) with A1‐like (*Serping1, Ggta1, H2‐D1, H2‐T23*) and A2‐like (*Tgm1, Clcf1, S100a10*) related markers. G) Simplified illustration of the Kynurenine catabolism with neuroprotective and neurotroxic branches metabolites. H–I) Kynurenine H) and Kynurenic acid (KYNA) I) amounts in unconditioned culture media (Medium), in non‐injured slices (CTR) conditioned media, in TBI slices conditioned media, in medium + SYNT, and in treated TBI slices conditioned media (TBI SYNT). Data are presented as mean ± SEM from at least 2 independent experiments, n = 6–8 each. One‐way ANOVA, followed by Tukey post‐test. *p < 0.05, **p < 0.01, ***p < 0.001.

Based on the identified metabolites (Figure 2E), we formulated a synthetic cocktail (SYNT). SYNT efficacy was tested in our recently developed in vitro 3D TBI model using organotypic brain slices^[^ ^13^ ^]^ (**Figure** **3**A). Cortical slices were subjected to TBI, and SYNT was administered 1 h after injury. SYNT treatment significantly reduced cell mortality in the lesion core as shown by PI staining (Figure 3B,C); neuronal protection was confirmed by decreased neurofilament light chain (NfL) release (Figure 3D). Notably, reducing PGs and KYN concentration to one‐tenth of the original dose (0.1x) abolished the protective action of the SYNT formulation, whereas both 1x and 10x doses significantly reduced cell death, with no added benefit at the higher dose (Figure 3E). These results support the safety of SYNT at elevated doses. When testing the effect of the single components, neither prostaglandins nor kynurenine alone significantly reduced cell death, indicating a synergistic effect when combined (Supplementary Figure S4, Supporting Information)

Gene expression analyses confirmed SYNT‐induced neuroprotection, as observed by the reversal of the TBI‐induced mRNA downregulation of *NeuN* and *BDNF* (Figure 3F). In addition, SYNT treatment shaped microglial response, reducing its activation (as for *CD11b* pan‐marker expression), and the pro‐inflammatory markers *IL‐6* and *CD86*, while upregulating the anti‐inflammatory marker *Arginase1* (Figure 3F), thus confirming its polarization toward a protective phenotype. When analyzing astrocyte related markers, a similar upregulation of the pan‐marker *GFAP* was found in TBI and TBI SYNT slices; among the previously classified A1 (*Serping1, Ggta1, H2‐D1, H2‐T23*) and A2 (*Tgm1, Clcf1, S100a10*) markers,^[^ ^14^ ^]^ a significant *Ggta1* upregulation concomitantly with a *H2‐D1* and *H2‐T23* down‐regulation were found in TBI SYNT. These results suggest that SYNT exerts a less clearly defined influence on astroglial activation (Figure 3F).

Given the role of kynurenine in neuroprotection or neurotoxicity depending on the activation of their distinct catabolic branches,^[^ ^15^, ^16^ ^]^ we examined the kynurenine metabolites released in the medium after TBI and SYNT treatment (Figure 3G). In the culture media of CTRL or TBI slices, the levels of kynurenine were similar to those of NB medium, indicating that cortical slices do not release kynurenine in culture conditions (Figure 3H). SYNT treatment (medium + SYNT), as expected, contained high amount of kynurenine. However, a significant uptake was found in the TBI + SYNT group (Figure 3H), indicating the induction of kynurenine catabolism. Notably, the protective metabolite kynurenic acid (KYNA) increased in the TBI + SYNT group (Figure 3I) while the neurotoxic metabolite 3‐hydroxyanthranilic acid (3HAA) remained undetectable, indicating a shift toward the protective branch of the pathway.

### 2.4 CM and SYNT Treatments Induce Chronic Protection up to 6 Months Post Injury

**Figure 4:**
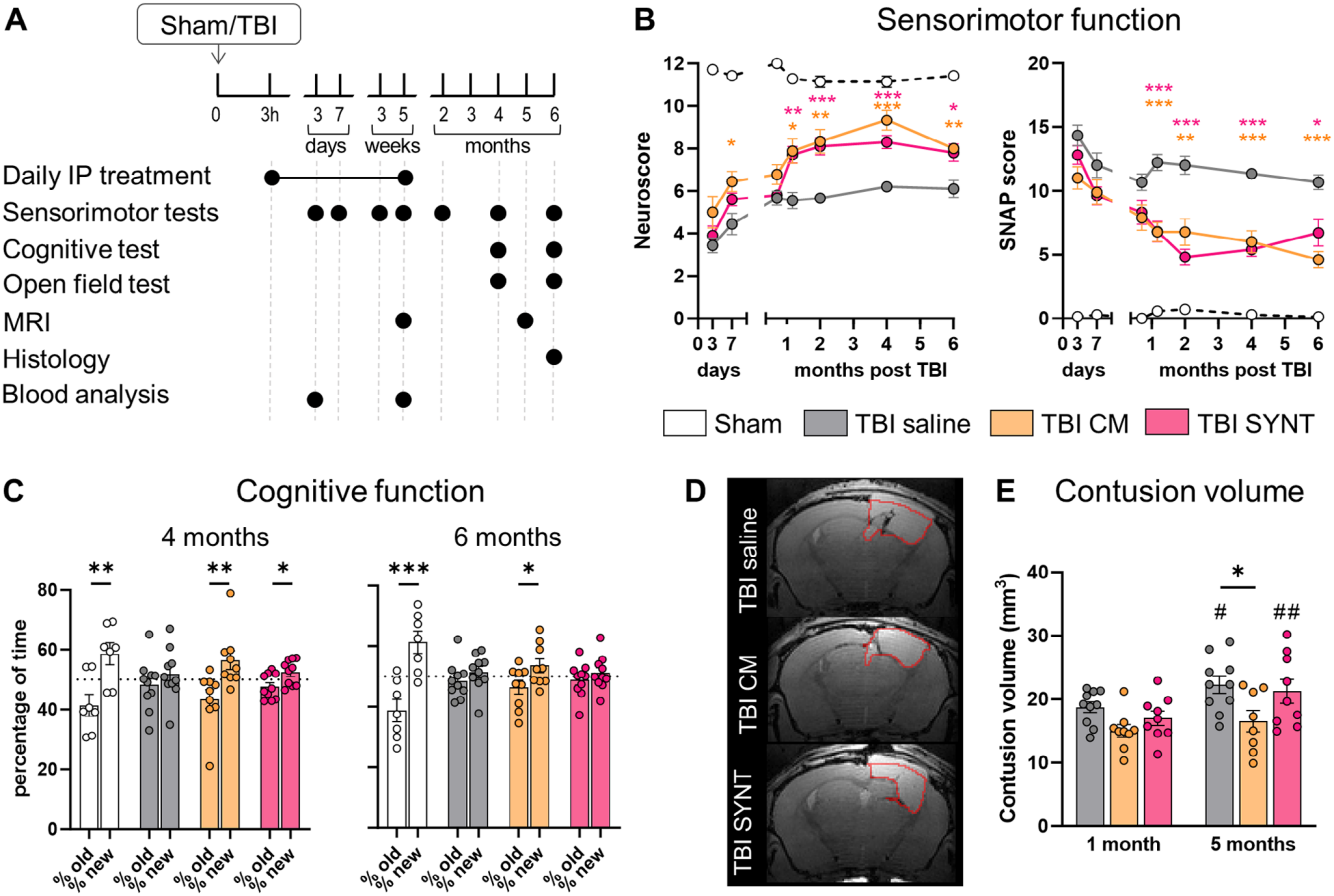
Chronic effects of CM and SYNT treatments after TBI in vivo. A) Schematic representation of the experimental design of Cohort 4 aimed at defining the long‐term efficacy of CM and the synthetic cocktail in TBI mice. B) Longitudinal sensorimotor assessment up to 6 months post‐injury by Neuroscore and Simple Neuroassessment of Asymmetric imPairment (SNAP). C) Cognitive assessment at 4‐ and 6‐months post TBI by Y maze test. D) Representative images of MRI acquisition with definition of lesion areas. E) Quantification of contusion volume at 1‐ and 5‐months post‐injury. Data are presented as mean ± SEM; n = 8–10 mice/group. B, E) Two‐way ANOVA for repeated measurement, followed by Tukey post‐test. C) *t*‐test. *p < 0.05, **p < 0.01, ***p < 0.001; #p<0.05, ##p<0.01, 1‐month versus 5‐months post TBI among the same treatment group.

We next tested SYNT in vivo, comparing its effects to total CM treatment (Cohort 4). TBI mice were treated daily for five weeks with saline, CM, or SYNT as in Cohort 1. Then treatments were held, and mice were followed up to 6 months (**Figure** **4**A). Both CM and SYNT demonstrated a favorable safety profile. Body weight trajectories up to 6 months post‐injury (Supplementary Figure S5A, Supporting Information) showed expected TBI‐related reductions in weight gain, but no differences between treated and untreated groups, indicating no treatment‐induced systemic toxicity. Similarly, spleen weights at sacrifice were comparable across groups (Supplementary Figure S5B, Supporting Information), excluding signs of splenomegaly or chronic inflammation.^[^ ^17^ ^]^ Moreover, we performed plasma biochemical analyses covering markers of general health (e.g., total protein, albumin, glucose), kidney (e.g., blood urea nitrogen ‐BUN‐, creatinine), liver (e.g., aspartate aminotransferase ‐AST‐, bilirubin), and heart/muscle (creatine kinase) function. All parameters were within normal ranges and showed no significant differences between groups, confirming the safety of repeated CM and SYNT administration (Supplementary Figure S5C–P, Supporting Information).

Both CM and SYNT significantly improved sensorimotor function, with persistent effects up to 6 months post‐injury (Figure 4B). A clear improvement in cognitive performance in the Y‐maze test was observed in both CM‐treated and SYNT‐treated mice (Figure 4C), but persisted at 6 months only in CM‐treated mice. Open Field tests at 4 and 6 months showed no group differences in locomotor activity. TBI animals displayed increased anxiety‐like behavior (more time in the periphery), which was not affected by CM or SYNT treatment (Supplementary Figure S1C–D, Supporting Information).

MRI analysis revealed that while contusion volume increased over time in TBI saline group, CM treatment prevented the progressive enlargement of the contusion, resulting in significantly less anatomical damage at 5 months (Figure 4D,E). This effect was not present after SYNT treatment (Figure 4D,E).

### 2.5 CM and SYNT Treatments Induce Chronic Modulation of Glial Activation

**Figure 5:**
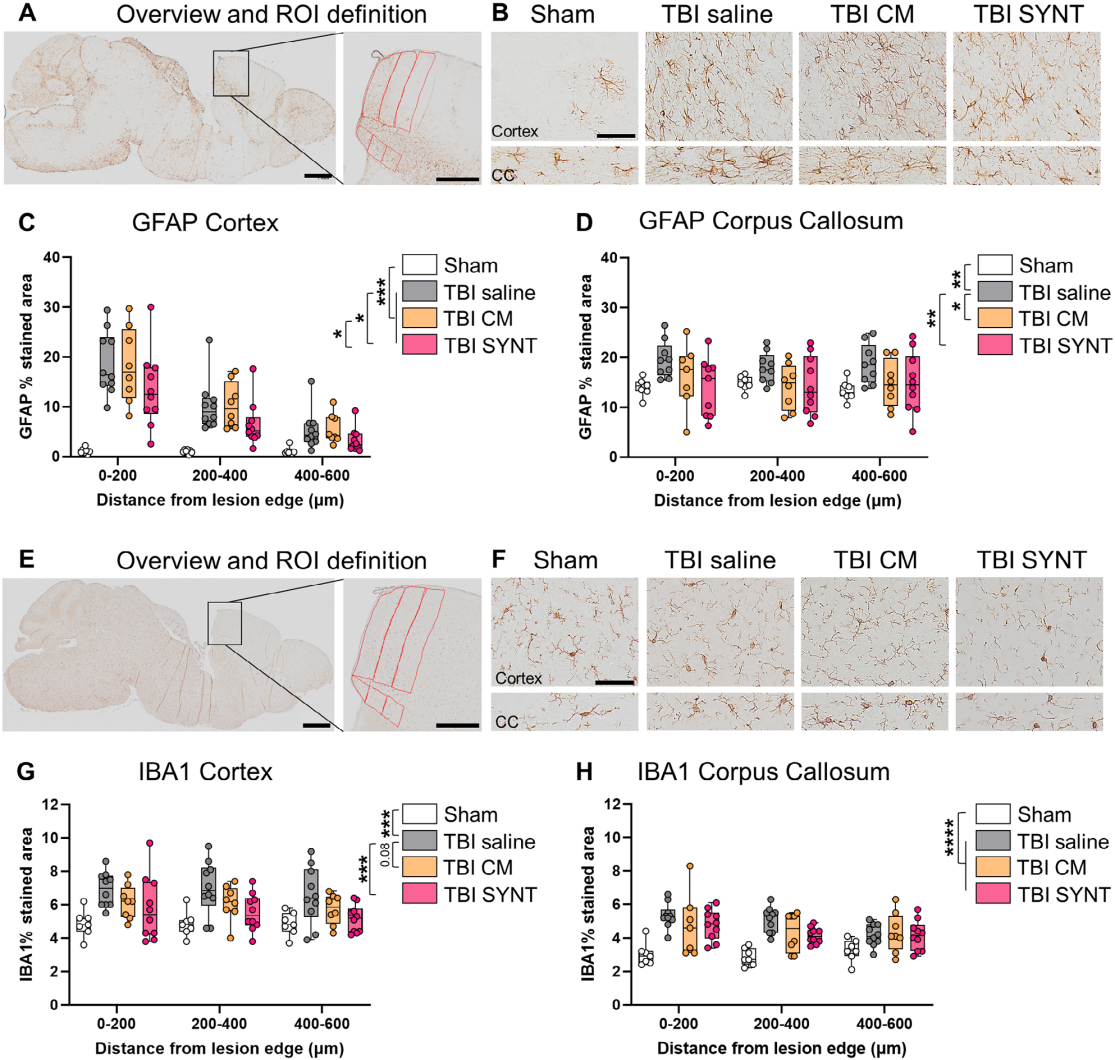
Glial activation 6 months post‐TBI. A) Overview and region of interest (ROI) definition of GFAP staining. B) Microphotographs showing GFAP staining in the pericontusional tissue of TBI saline, TBI CM, and TBI SYNT groups mice, 6‐month post‐injury. C,D) Quantification of GFAP staining in the cortex and in the corpus callosum. E) Overview and ROI definition of IBA1 staining. F) Microphotographs showing IBA1 staining in the pericontusional tissue of TBI saline, TBI CM, and TBI SYNT groups mice, 6‐month post‐injury. G,H) Quantification of GFAP staining in the cortex and in the corpus callosum. Data are presented as median + interquartile range (box) with whiskers showing min and max values n = 8–10. Data are analyzed by Two‐way ANOVA followed by Tukey post‐test (on main effect). *p < 0.05, **p < 0.01, ***p < 0.001. Bar on A–E = 1 mm, inserts = 500 µm, B–F = 50 µm.

At 6 months post‐injury, both CM and SYNT reduced astrogliosis in the pericontusional cortex and corpus callosum, with SYNT showing a more pronounced effect in reducing GFAP staining (**Figure** **5**A–D). CM and SYNT also decreased microglial activation in the pericontusional cortex compared to saline (Figure 5E–G), although no significant treatment effect was observed in the corpus callosum (Figure 5H).

### 2.6 CM and SYNT Treatments Induce Protective Changes of Circulating Factors

**Figure 6:**
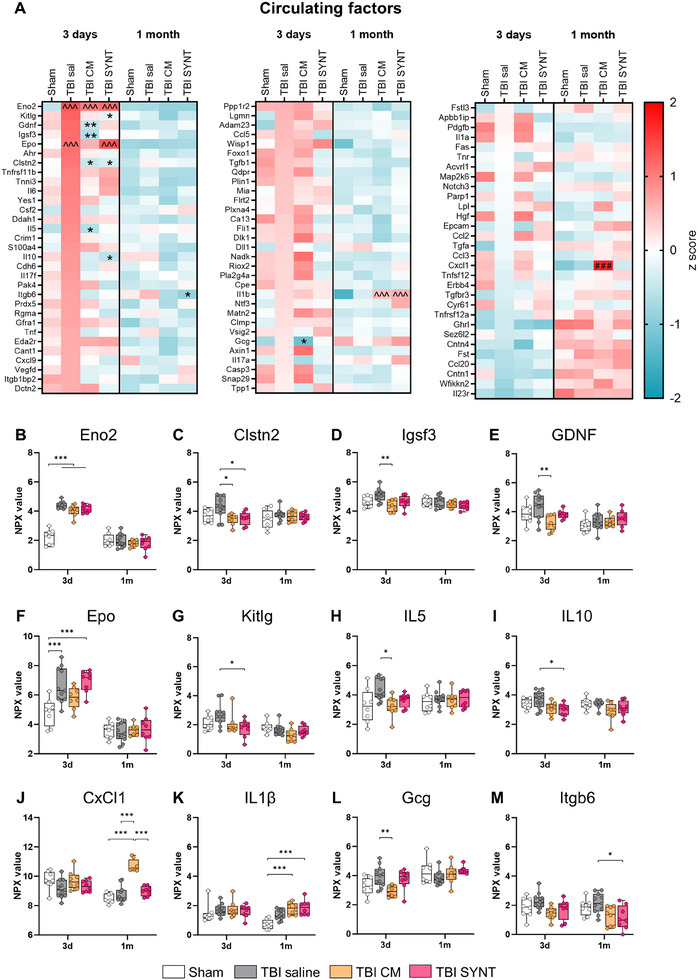
Circulating factors at 3 days and 1 month post‐injury. A) Heatmap based on Z‐score values for the 92 measured proteins. B–K) Graphs show the amount (as NPX value) of the circulating proteins differentially expressed among groups. Data are presented as median + interquartile range (box) with whiskers showing min and max values n = 8–10. B–M) Linear random intercept regression, with *p*‐values adjusted by Benjamini & Hochberg correction for multiple comparisons. A) ^*p*<0.05, ^^^*p*<0.001 versus Sham; *p < 0.05, **p < 0.01 versus TBI saline; ### *p*<0.001 TBI CM versus all other groups.

Targeted proteomic analysis of plasma samples at 3 days and 1 month post‐TBI revealed distinct alterations in circulating proteins involved in neuronal function, inflammation, and cell proliferation by TBI, and CM or SYNT treatments (**Figure** **6**A). At 3 days post‐injury, a significant increase of Eno2 (also known as neuron‐specific enolase NSE) was observed, indicating acute neuronal damage. However, this increase was unaffected by either treatment (Figure 6B). Instead, both CM and SYNT led to a significant reduction in the synaptic protein Calsyntenin‐2 (Clstn2) levels compared to saline, with CM treatment selectively reducing the axonal protein Igsf3 (Figure 6C,D) and glial‐derived neurotrophic factor (GDNF, Figure 6E), underscoring its neuroprotective potential. Epo (erythropoietin, Figure 6F) and Kitlg (the ligand for the receptor‐type protein‐tyrosine kinase KIT, Figure 6G), both involved in the stimulation and differentiation of hematopoietic stem/precursor cells, were reduced by CM and SYNT respectively. Inflammatory mediators, including IL‐5 and IL‐10, were also decreased by CM and SYNT, respectively (Figure 6H,I), while CM uniquely increased Chemokine Ligand 1 (CxCl1) at 1 month (Figure 6J) and both CM and SYNT increased IL1β (Figure 6K). Additionally, the neuroendocrine factor glucagon (Gcg) was acutely reduced by CM treatment (Figure 6L), while integrin beta‐6 (Itgb6), which is involved in extracellular matrix interactions, was selectively lowered by SYNT at 1 month (Figure 6M). These findings suggest that CM provides a broad range of neuroprotective and inflammatory modulating effects, while SYNT replicates some of these effects, thought to a lesser extent.

## 3 Discussion

In the present study, we demonstrated the neuroprotective effects of hAMSC‐secretome treatment in experimental in vitro and in vivo TBI mouse models. We identified kynurenine and prostaglandins (PGE2, PGJ2, and PGA2) as key bioactive components within the secretome. When administered as part of the SYNT, these metabolites conferred significant long‐term functional benefits, improving sensorimotor function up to 6 months and memory at 4 months post‐injury. While SYNT's composition can be further optimized, this study provides the first evidence of lasting therapeutic effects from a synthetic formulation based on secretome‐released factors. Notably, only the complete CM reduced contusion volume at 5 months, highlighting the potential added value of the full secretome.

Building on our previous work using an in vitro model of acute brain injury, where we demonstrated comparable efficacy between hAMSCs and their CM,^[^ ^12^ ^]^ we first verified the effectiveness of CM in vivo after experimental TBI. Daily systemic infusions of CM starting 3 hours post‐injury, led to significant functional protection, improving sensorimotor and cognitive functions. At the molecular level, CM treatment triggered acute changes in the brain microenvironment, including upregulation of genes involved in ECM remodeling. These findings suggest a shift toward a microenvironment that may limit fibrosis^[^ ^18^ ^]^ and promote restorative plasticity, potentially through the reduction of perineuronal nets, as previously observed in stroke models treated with MSC,^[^ ^19^ ^]^ and through enhanced ECM degradation facilitating neovascularization.^[^ ^20^ ^]^ Consistent with this interpretation, we observed at one month post‐injury an increased neuronal and vascular density in the pericontusional cortex and reduction of anatomical damage aligning with prior studies on CM efficacy in TBI.^[^ ^21^, ^22^, ^23^ ^]^ Notably, control experiments with the unconditioned culture medium (NB) showed no improvement in functional outcomes, underscoring the specificity of the protective effects of hAMSC‐released factors. When assessing central (ICV) delivery, we observed significant improvement of sensorimotor function, but no effects on contusion volume,^[^ ^24^ ^]^ suggesting that single central delivery may be insufficient and that repeated dosing may be required. Given reduced invasiveness, systemic delivery stands as the preferred therapeutic route.

### 3.1 Cell‐Free Therapy Potential: Focus on Bioactive Metabolites

With the aim of developing a cell‐free therapy, we tested the effects of the fraction of CM enriched with small (<700 Da) metabolites previously identified as protective in vitro.^[^ ^12^ ^]^ The efficacy of this metabolite‐enriched fraction in vivo, following TBI, validated the metabolomic screening of its composition. Metabolomic analysis of three donor pools (each from >10 donors) revealed an enrichment of four key metabolites in the protective fraction: three prostaglandins and kynurenine. These metabolites were then formulated into a synthetic cocktail (SYNT), which, when tested in our in vitro TBI model,^[^ ^13^ ^]^ demonstrated neuroprotective effects, including reduced neuronal death and lower release of NfL, a marker of neuronal injury, in the culture media. Gene expression analysis further confirmed neuroprotection, revealing the reversal of TBI‐induced downregulation of NeuN and BDNF, and a modulation of microglial activation enhancing anti‐inflammatory and tissue repair traits.

### 3.2 Long‐Term Efficacy of CM and SYNT in TBI Recovery

Both CM and SYNT produced long‐term functional improvements in the in vivo TBI model, with sensorimotor performance enhanced up to 6 months post‐injury, and cognitive function significantly improved by 4 months. Importantly, previous findings on CM efficacy after TBI have only examined shorter time frames, up to 1 month post‐injury.^[^ ^21^, ^22^, ^23^ ^]^ This study is the first to demonstrate the long‐term persistence of the therapeutic effects of both CM and SYNT, even after discontinuing treatment, indicating that early intervention with these treatments can shift the injury trajectory toward better outcomes. Histological analysis revealed sustained neuroprotection, with reduced neuroinflammation, decreased microglial activation, and attenuated astrogliosis observed in the pericontusional region at 6 months post‐injury. These findings are consistent with studies showing the ability of prostaglandins and kynurenine to modulate immune responses and promote neuroprotection as detailed below.

### 3.3 Mechanisms of Neuroprotection: Prostaglandins and Kynurenine Pathways

Bioactive factors from CM can directly reach the brain by active blood‐brain barrier (BBB) transport (as for KYN)^[^ ^25^ ^]^ or following BBB disruption or leakage associated with TBI^[^ ^26^, ^27^ ^]^ and also regulate a variety of proteins related to neuroprotection in plasma. Thus, we discuss how PGs and KYN systemic and brain local effects contribute synergistically to the observed neuroprotection.

While PGE2 was traditionally associated with pro‐inflammatory effects, it is increasingly recognized that it also plays nuanced and context‐dependent dual roles in inflammation and neuroprotection. Indeed, when binding its cognate E‐prostanoid receptors (EP1‐EP4),^[^ ^28^, ^29^ ^]^ PGE2 could activate downstream pathways associated with toxic (EP1, EP3)^[^ ^30^, ^31^ ^]^ or protective (EP2, EP4)^[^ ^32^, ^33^ ^]^ effects after acute brain injury.

At cellular level, it has been demonstrated that PGE2 inhibits microglial activation after TBI.^[^ ^34^ ^]^ Notably, the amounts of MSC‐released PGE2 mirrored the immunomodulatory action on microglia, suggesting PGE2 as a potential marker of MSC potency.^[^ ^34^ ^]^ PGE2 also modulates LPS‐activated astrocytes by reducing TNFα release and promoting the expression of neurotrophic factors such as BDNF and NT‐3.^[^ ^35^ ^]^ PGJ2 acting as a PPAR‐γ agonist, inhibits microglial‐induced neurotoxicity.^[^ ^36^ ^]^ In an in vitro model of neuron‐microglia co‐cultures, PGJ2 binding to PPAR‐γ inhibits microglial pro‐inflammatory activation (following LPS/IFN‐γ stimulation) and mitigates reactive‐microglia‐induced neurotoxicity via a CD200‐CD200R1 dependent mechanism.^[^ ^37^ ^]^ Furthermore, chronic administration of PPAR‐γ following repeated mild TBI improves cognition at 6 months and is associated with reduced microglial and astrocytic activation in the cortex, hippocampus, and white matter, alongside a dampening of microglia‐associated inflammatory pathways, particularly IL‐6 and TNFα production.^[^ ^38^ ^]^ Indeed, prostaglandins have been implicated in the modulation of immune cells, with PGE2 inhibiting T and B cell proliferation,^[^ ^39^, ^40^, ^41^ ^]^ promoting T regs^[^ ^42^ ^]^ and inducing macrophage polarization toward an anti‐inflammatory, pro‐healing phenotype.^[^ ^5^, ^43^, ^44^, ^45^ ^]^ Notably, we have multiple pieces of evidence confirming the immunomodulatory properties of PGE2 in the hAMSC‐derived CM,^[^ ^40^, ^41^, ^45^ ^]^ used in the present study. Indeed, CM devoid of prostanoids, obtained by culturing hAMSC with the cyclooxygenase inhibitor indomethacin, was only partially able to block T cell proliferation,^[^ ^40^ ^]^ B cell proliferation,^[^ ^41^ ^]^ and M1 differentiation.^[^ ^45^ ^]^

Kynurenine, a tryptophan metabolite, exerts immunomodulatory effects by inhibiting T cell proliferation, and reducing TNFα production by monocytes and microglia under inflammatory conditions both in vitro and in vivo.^[^ ^46^, ^47^, ^48^ ^]^ It also has direct neuroactive effects,^[^ ^15^ ^]^ with dual neuroprotective and neurotoxic actions depending on its metabolites. Neurotoxicity is primarily associated with QUIN, a potent NMDA receptor agonist that can inhibit the astrocytic glutamate reuptake, perpetuating neurotoxicity,^[^ ^16^ ^]^ while also promoting ROS generation, BBB disruption, tau phosphorylation and cytoskeleton destabilization.^[^ ^49^ ^]^ Conversely, KYNA, acting as an NMDA and α7‐nicotinic‐acetylcholine receptor antagonist, confers protection against excitotoxic damage.^[^ ^50^, ^51^ ^]^ More recently, KYNA has been identified as an agonist of the orphan G‐protein‐coupled receptor GPR35, which modulates cAMP production and inhibits N‐type Ca^2^⁺ channels in sympathetic neurons and astrocytes, ultimately suppressing several inflammatory pathways.^[^ ^52^ ^]^ KYN can pass the intact BBB, transported by the large neutral amino acid transporter (LAT1).^[^ ^25^ ^]^ At brain level, production of KYNA is mainly associated to astrocyte activation, due to their expression of kynurenine aminostransferases (KATs), while neurotoxic metabolites are associated to pro‐inflammatory microglia activation, due to its expression of kynurenine 3—monooxygenase (KMO).^[^ ^53^ ^]^ In our in vitro model, we observed a SYNT‐induced dampening of microglia activation while astrocytes were clearly up‐regulated after TBI. These effects were associated to a clear preference for the neuroprotective branch after SYNT treatment, supporting its role in attenuating TBI‐induced damage. In the present study, we demonstrated the SYNT ability to acutely shape microglial response in vitro, decreasing its overall activation and pro‐inflammatory markers, while inducing an up‐regulation of *Arginase1* marker, associated with anti‐inflammatory phenotype and tissue repair. Of note, SYNT exposure, induced a downregulation of genes associated with antigen presentation (*CD86, H2‐D1, H2‐T23*), in compliance with the immunoregulatory properties of MSC and MSC derivatives. Notably, administration of single factors did not confer protection, highly suggesting a synergistic effects of the SYNT. Furthermore, our proteomic data in plasma discussed below suggest that a synergistic systemic and brain‐local action is responsible for the observed neuroprotection.

### 3.4 Insights from Blood Proteomics into CM and SYNT Effects

Targeted proteomic analysis of plasma proteins revealed a treatment‐induced reduction of Calsyntenin‐2 (Clstn2), a protein involved in synaptic function and inhibitory transmission in the brain^[^ ^54^ ^]^ and of the immunoglobulin superfamily member 3 (Igsf3), a protein of the axonal terminal involved in neuronal morphogenesis.^[^ ^55^ ^]^ These data support acute neuroprotective effects of both CM and SYNT. We also observed treatment‐induced decreases (with slightly different effects between CM and SYNT) in plasma levels of Epo and Kitlg, both of which are involved in hematopoietic progenitor cell proliferation and differentiation, and are associated to inflammatory diseases.^[^ ^56^ ^]^ The reduction of these proteins by CM and SYNT supports their immunomodulatory roles, potentially reducing the activation and infiltration of immune cells, that contribute to a harmful neuroinflammatory environment and worsening of brain damage.^[^ ^57^, ^58^, ^59^ ^]^ Additionally, we found reduced levels of GDNF, and both pro‐inflammatory IL5 and anti‐inflammatory IL10 cytokines, aligning with a broader dampening of the inflammatory response during the critical post‐injury phase. Interestingly, CM induced a selective increase in CxCl1 at 1 month post‐injury, which likely reflects a role in angiogenesis and wound healing^[^ ^60^, ^61^ ^]^ rather than a role in neutrophil recruitment^[^ ^62^ ^]^ typically associated with this chemokine acutely after injury. This aligns with our histological findings of increased vessel density in pericontusional tissue at five weeks and is consistent with MSC‐secretome‐induced endothelial cell proliferation in vitro.^[^ ^63^ ^]^ Interestingly, the TBI CM and TBI SYNT groups, but not the TBI saline group, showed a chronic increase in IL‐1β compared to the sham group. While IL‐1β is generally linked to pro‐inflammatory responses and is considered detrimental in the acute phase post‐injury, evidence from our group and others suggests that IL‐1β is associated with microglia/macrophage activation toward a pro‐resolutive phenotype following MSC treatment in TBI models^[^ ^64^ ^]^ and spinal cord injury.^[^ ^65^ ^]^ Additionally, IL‐1β has been shown to potentiate neurotrophic effects on neurite growth.^[^ ^66^ ^]^

We also observed a CM‐induced decrease in glucagon (Gcn) acute plasma levels. Following TBI, the brain undergoes significant metabolic changes, including an energy crisis caused by disrupted blood flow, mitochondrial damage, and impaired glucose metabolism, which increases glucose demand.^[^ ^67^ ^]^ Glucagon promotes glycogenolysis and gluconeogenesis, helping to ensure an adequate glucose supply to the brain.^[^ ^68^ ^]^ A reduction in circulating glucagon may indicate a less severe metabolic disruption and energy imbalance induced by hAMSC‐secretome treatment. Lastly, we found a reduction in Itgb6 in the TBI SYNT group. Plasma elevation of Itgb6 has been proposed as a novel serum tumor marker,^[^ ^69^ ^]^ but its relevance in acute brain injury warrants further investigation.

### 3.5 Limitations and Future Directions

A potential limitation of our study is the reliance on in vitro models for the initial selection of protective mediators, which may not fully capture the complexity of TBI pathology. As such, additional bioactive factors of protein or nucleic acid origin, potentially contributing to the neuroprotective effects of MSC‐derived secretome,^[^ ^70^ ^]^ may have been overlooked. Of note, while SYNT showed promising neuroprotective effects, it did not replicate all the long‐term benefits observed with total CM, particularly in cognitive function and chronic anatomical damage. This suggests that other mediators present in the total CM may contribute to its superior protective potential. Indeed, other groups have identified growth factors such as hepatocyte growth factor (HGF),^[^ ^71^, ^72^ ^]^ transforming growth factor β (TGF‐ β)^[^ ^73^ ^]^ or other inflammatory mediators such as TNF stimulated gene‐6 (TSG‐6),^[^ ^74^, ^75^, ^76^ ^]^ stromal cell‐derived factor‐1 (SDF‐1),^[^ ^77^ ^]^ as MSC‐derived mediators of protection after acute or chronic neurologic conditions. In addition, our previous omics analysis revealed the presence of several miRNA contained in extracellular vesicles (EV) of total CM.^[^ ^78^, ^79^ ^]^ Among the various miRNAs identified, some possess neuroprotective and anti‐inflammatory effects (i.e., miR‐21‐5p, miR‐22‐5p, and miR‐146a‐5p),^[^ ^80^ ^]^ which may explain several of the effects herein observed. Specifically, miRNA‐146a has been shown to influence M2 macrophage polarization,^[^ ^81^ ^]^ modulate TLR and cytokine signaling,^[^ ^82^ ^]^ and regulate neurotrophic factors such as GDNF.^[^ ^83^ ^]^ In addition, miRNA‐146a and miR‐21‐5p modulate the NF‐κB transcription factor,^[^ ^84^ ^]^ which in turn is implicated in glucagon (Gcg) signaling.^[^ ^85^ ^]^ Interestingly, our proteomics analysis revealed that both GDNF and Gcg were significantly reduced in the blood of CM‐treated, but not SYNT‐treated, mice. Therefore, future studies should focus on identifying these additional components to optimize the composition of the synthetic cocktail.

Interestingly, early studies have shown that acute administration of KYNA after TBI attenuates cognitive deficits, improves neurological motor function, and reduces localized edema formation and hippocampal neuronal loss.^[^ ^86^, ^87^ ^]^ However, in other pathological contexts, such as cardiac arrest, complete inhibition of the kynurenine pathway in IDO‐/‐ mice markedly improved survival and neurological outcomes, while administration of KYN to IDO‐/‐ mice reversed these protective effects.^[^ ^88^ ^]^ The mechanisms driving kynurenine toward neuroprotective or neurotoxic pathways, and whether specific pathology‐dependent triggers are involved, require further investigation

## 4 Conclusion

Our study provides first evidence of the neuroprotective effects of hAMSC‐secretome treatment in TBI that improve disease trajectories, decreasing neurodegenerative processes, and improving recovery up to 5 months post‐TBI in mice. Through comprehensive metabolomic analysis, we identified four metabolites selectively enriched in the protective fraction, and we successfully reconstructed them into a synthetic cocktail with significant therapeutic potential persisting chronically after TBI. Although further optimization is needed to match the efficacy of the full secretome, our findings lay the groundwork for developing a fully characterized and standardized cell‐free therapy based on hAMSC‐derived components for treating TBI.

## 5 Experimental Section

### MSC‐Secretome Collection

Human term placentas (*n*>45) were provided by the Department of Obstetrics and Gynecology of Fondazione Poliambulanza‐Istituto Ospedaliero of Brescia, Italy. Samples were collected following informed written consent as indicated by the Comitato Etico Provinciale of Brescia, Italy number NP 2243 (19/01/2016).

### hAMSC‐Secretome was Collected As Conditioned Media (CM) as Previously Described^[^ ^12^, ^89^ ^]^

Cells were cultured for 5 days at 37 °C in 5% CO_2_ in NB supplemented with B27 (NB/B27, B27 1:50; L‐glutamine, 1:100; penicillin, 100 U mL^−1^; streptomycin, 100 µg mL^−1^). After culture, CM was collected, centrifuged at 300 × g, filtered (0.8 µm), and stored at −80 °C. To avoid the possibility of a donor‐related variability, three different CM batches, each corresponding to a pool of more than 10 donors, were used for the study. Negative controls are CM from human dermal fibroblasts, which we previously demonstrated to have a non‐protective role in our in vitro model of acute brain injury^[^ ^12^ ^]^ or unconditioned media (the NB/B27 medium cultured for 5 days at 37 °C in 5% CO_2_).

### CM‐Fractionation

Sequential size‐exclusion/gel‐filtration chromatography (Vivaspin2 column, Sartorius Stedim; PD MiniTrapG‐10, GE Healthcare Life Sciences) was used as previously described.^[^ ^12^ ^]^ Briefly, CM was loaded into a 2 kDa cut‐off column, centrifuged at 3200 g for 30 min at RT. The resulting CM<2 kDa fraction was further fractionated using PD MiniTrap G‐10 gel‐filtration, collecting fractions Fr>700 Da and Fr<700 Da, which respectively resulted non‐protective and protective in our in vitro model of acute brain injury.^[^ ^12^ ^]^

### Metabolites Extraction

CM fractions <2 kDa, >700 Da, and <700 Da with their negative control counterparts were lyophilized up to 50 µL. Three different preparations/conditions were submitted to untargeted and targeted metabolomics approaches.

### Untargeted Metabolomics (FIA‐HRMS)

FIA‐HRMS was used for untargeted metabolomics.^[^ ^90^ ^]^ Eight µL of each extract was analyzed using the QExactive Mass Spectrometer (Thermo Fisher Scientific) equipped with an electrospray source operated in negative and positive modes. Each run was carried out at a flow rate of 50 µL min^−1^ (Agilent 1200 Series) of mobile phase consisting of isopropanol/water (60:40, v/v) pH 9 (5 mM ammonium) for negative mode and methanol/water (60:40, v/v) pH 3 (0.1% formic acid) for positive mode. Reference masses (m/z 210.1285 for positive and m/z 212.0750 for negative ionization) for internal calibration were infused during the analysis. Mass spectra were acquired from m/z 50 to 1000 with 60 000 resolutions. Source temperature was set to 240 °C with 25 L min^−1^ drying gas and a nebulizer pressure of 35 psig. MS/MS fragmentation pattern of the significant features was collected and used to confirm metabolite identity. All data processing and analysis were done with EASY‐FAA using an in‐house‐developed script.^[^ ^90^ ^]^

### Targeted Metabolomics p180 Biocrates Kit

A targeted quantitative approach using the AbsoluteIDQ p180 kit (Biocrates) was applied as previously published.^[^ ^91^ ^]^ The method combines derivatization and extraction of analytes with the selective mass‐spectrometric detection using multiple reaction‐monitoring pairs. The p180 kit allows simultaneous quantification of 186 metabolites (40 amino acids and biogenic amines, 40 acylcarnitines, 90 glycerophospholipids (lysophosphatidylcholines, lysoPCs, phosphatidylcholines, PCs, sphingomyelins SMs), 15 sphingomyelins, 1 monosaccharide).

### Targeted Metabolomics Central Metabolism (LC‐MRM)

The assay monitored the abundance of 83 metabolites divided in amino acids and derivatives (32), nucleic acid‐related compounds (19), vitamins (13), sugar (4), and other metabolites belonging to glycolysis, TCA cycle (18). Metabolites were extracted using cold MeOH (1:4), incubated 20 min at −80 °C, centrifuged at 13 000 g X 15 min. One µL of extract was injected into a Discovery HS F5‐3 (Sigma Aldrich) (2.1 mm I.D. × 150 mmL, 3 µm), using a 20 min gradient from 0 to 95% A (10 mm NH₄HCO₂ pH 3.5), B (Acetonitrile), at 350 µL min^−1^. The mass spectrometer was equipped with an ESI source operating in both positive and negative ions and selected reaction monitoring (SRM) mode. The transitions identified during the optimization of the method were reported in Supplementary Table S1 (Supporting Information). The MS settings were: nebulizing gas flow rate: 3.0 L min^−1^; drying gas flow rate: 15.0 L min^−1^; DL Temperature: 250 °C; block heater temperature: 400 °C. Peak areas were automatically integrated using LabSolution Insight LC MS (Shimadzu). Retention times and area ratio between quantifier and qualifier ions of all metabolites were confirmed using pure standards.

### Lipid Mediators

CM fractions <2 kDa, >700 Da, <700 Da, and their control counterparts (NB) were thawed, and deuterium‐labelled internal standards (LXA4‐d5, 5‐HETE‐d8, LB4‐d5, MaR2‐d5, RD2‐d5, RE1‐d4, AA‐d11, EPA‐d5, LXA4‐d5) were added. Each sample was subjected to solid phase extraction (Isolute SPE, Biotage), collecting methyl formate fraction. Following solvent evaporation, extracts were resuspended in methanol/water (1:1, vol/vol) and injected in a Shimadzu LC8060 mass spectrometer using a Kinetex C8 column (150 mm × 2.1 mm × 2.6 µm) at 40 °C using a 0.4 mL min^−1^ flow rate (A 0.1% formic acid, B acetonitrile) over 25 min. Instrument operated in a multiple reaction monitoring (MRM) method and positive and negative acquisition mode. The transitions identified during the optimization of the method were reported in Supplementary Table S2 (Supporting Information). Each lipid mediator was identified by comparison with retention time of authentic standards. Calibration curves were obtained for each mediator using lipid mediator mixtures from 4 to 150 pg µL^−1^ that gave linear calibration curves with an R^2^ values of 0.98–0.99.

### Metabolite Selection

Metabolites enriched in the CM protective fractions (<2 kDa, <700 Da) were selected following the decision tree illustrated in Figure 2D. Specifically, fold changes in abundance were calculated for all metabolites across all comparisons. At each step of the decision tree, only metabolites with a fold change consistent with the criteria, advanced to the next stage of evaluation.

### Synthetic Cocktail

For in vitro experiments, SYNT (1×) was reconstituted based on the metabolomic analysis of CM fraction <2 kDa, using the highest measured concentrations of bioactive components: PGA2 (10 ng/mL), PGE2 (200 ng mL^−1^), PGJ2 (1 ng mL^−1^) (Cabru, #10 210, #14 010, #18 500), and kynurenine (36 µm; Sigma, K8625). Prostaglandins were handled on dry ice and with glass instruments to prevent degradation.

Dose–response studies showed that protection was lost below 0.1× and remained stable up to 10× concentrations; therefore, the lowest effective concentration (1×) was selected for in vitro use.

For in vivo administration, SYNT was prepared at 2× concentration in sterile saline and stored at −80 °C for a maximum of two weeks before use. The concentration was doubled to compensate for potential dilution or reduced local bioavailability following systemic injection, ensuring effective exposure at the target site.

### Animals and Study Approval

C57BL/6J mice (adult male mice or pregnant female, ENVIGO) were housed in a specific pathogen‐free vivarium at a constant temperature (21 ± 1 °C) and relative humidity (60 ± 5%), with a 12 h light–dark cycle and ad libitum access to food and water.

The Istituto Ricerche Farmacologiche Mario Negri IRCCS maintains strict adherence to the regulations, laws, and policies which govern the use and care of laboratory animals: Italian Governing Law (D.lgs 26/2014; Authorisation n.19/2008‐A issued March 6, 2008 by the Italian Ministry of Health); additionally internal authorization for people conducting experiments on animals are provided by the Mario Negri Institutional Regulations and Policies (Quality Management System Certificate – UNI EN ISO 9001:2008 – Reg N° 8576‐A); EU guidelines and directives (EEC Council Directive 2010/63/UE) as well as the NIH Guide for the Care and Use of Laboratory Animals (2011 edition). Each was approved by the Mario Negri Institute Animal Care and Use Committee, which has ad hoc members included for ethical issues and by the Italian Ministry of Health (Decreto no. 435‐2017‐PR). The vivarium is maintained to international standards and is regularly inspected by a certified veterinarian who reviews procedures and experimental protocols, as well as monitoring the welfare of the animals.

All experiments were developed in accordance with Animal Research: Reporting of In Vivo Experiments (ARRIVE) guidelines,^[^ ^92^ ^]^ using biostatistics to optimize mouse numbers as in our previous work using the mouse TBI model^[^ ^93^, ^94^, ^95^ ^]^ and committing to refinement, reduction, and replacement to minimize the number of mice. Thus, for statistical validity, 6–10 mice were allotted for biochemical and biomarker analysis and 8–10 for behavioral testing. Researchers responsible for analyses performed were blinded to ensure an unbiased approach.

### Experimental Traumatic Brain Injury

Male mice, aged 2–3 months, received anesthesia via isofluorane (induction 4%, maintenance 1.5%) in a 70/30% mix of N_2_O/O_2_. Once anesthetized, they were placed in a stereotaxic frame with atraumatic ear bars inserted after application of local anesthetic (Emla) and eye lubrication (Lacrigel) in order to protect corneal membranes. As previously described, a craniotomy was performed with subsequent impact applied via the controlled cortical impact technique.^[^ ^87^ ^]^ The impact was performed using an electromagnetic piston (Impact One, Leica) which was mounted with a 3 mm diameter rigid impactor. The impact was performed at 20 degrees from the vertical axis in order to apply the force perpendicularly to the exposed dura mater at stereotactic coordinates from bregma antero‐posteriority −2.5 mm, medio‐laterality +2.5 mm. Impactor velocity was set to 5m s^−1^, deformation depth to 2 mm, and a dwell time of 0.1s in order to produce a severe traumatic injury.^[^ ^89^ ^]^ A cranioplasty was applied over the craniectomy site, and the scalp was sutured closed. During the entire procedure, a body temperature of 37 °C was maintained. Sham mice were not subjected to craniectomy nor brain injury, only to anesthesia, skin incision, and suturing.

### In Vivo Treatments

Mice were subjected to daily IP infusion (150 µL day^−1^) of placebo (saline or NB) or selected treatment (either CM or Fr<700 Da or SYNT) starting from 3 hours after injury up to sacrifice (for Cohorts 1–3) or up 5 weeks for long term experiments (Cohort 4).

A group of mice was treated with 5 µL of saline or CM intracerebroventricularly infused as previously described.^[^ ^64^ ^]^

### Behavioral Tests:Simple Neuroassessment of Asymmetric Impairment (SNAP)

Mice were evaluated using eight behavioral parameters, as previously described^[^ ^95^, ^96^ ^]^ to assess interaction with the handler, grip strength, visual placing, pacing or circling behavior, gait and posture, head tilt, visual field, coordination, and proprioception. Each test was scored on a scale from 0 (normal) to 5 (maximally impaired), with the total score ranging from 0 (indicating no impairment) to 40 (indicating severe impairment).

### Behavioral Tests:Neuroscore

Mice were evaluated on a scale from 4 (normal) to 0 (maximally impaired) across the following criteria: (1) forelimb function during grid walking and flexion response when suspended by the tail; (2) hindlimb function during grid walking and extension response during tail suspension; and (3) resistance to lateral pushes from both the right and left sides. The scores from each category were combined to produce a total score ranging from 0 (most impaired) to 12 (not impaired)^[^ ^97^, ^98^ ^]^

### Behavioral Tests: Y Maze

TBI‐induced spatial recognition memory deficits were assessed using the Y‐maze test as previously described.^[^ ^99^, ^100^ ^]^ During the first trial, each mouse was initially placed in one arm of the maze (designated as the “starting arm”) and allowed to explore only two accessible arms for 5 minutes. Following a 1‐hour inter‐trial interval, the mouse was returned to the same starting arm for a second 5‐minute session (trial 2, retrieval phase), this time with unrestricted access to all three arms of the maze. The time spent in each arm during both trials was recorded using EthoVision XT 15.0 (Noldus), and the % of time spent in the new and familiar arms was calculated.

### Open Field Test

General locomotor activity, exploratory, and anxiety‐like behaviors were assessed by the open field test. A gray Perspex square arena surrounded by walls (40 × 40 × 30 cm) with the floor divided into 25 squares (8 × 8 cm), placed in a specific room separated from the operator's room was used. The nine central squares (24 × 24 cm) represent the “center” and the surrounding border zone the “borders”. Mice were individually placed in the center of the arena for 5 minutes, and their movements were recorded by Ethovision XT, 15.0. Anxiety and exploratory‐related behaviors in the open field were assessed by quantifying the time spent in the “center” (inversely related to anxiety behavior) and in the “borders” (directly related to anxiety behavior) of the arena as previously described.^[^ ^19^ ^]^

### MRI Acquisition and Quantification

Anesthetized mice (induction: isoflurane 3%, maintenance: 1.5%, in a mixture of N_2_O/O_2_ (ratio: 70%/30%)), monitored for respiratory frequency and body temperature (37 °C) underwent imaging by 7T small‐bore animal scanner (BioSpec; Bruker) equipped with a quadrature 1H CryoProbe (Bruker) surface coil acting as transmitter and receiver and running ParaVision 6.01. A 3D fast low‐angle shot magnetic resonance imaging (FLASH) sequence (TR/TE 250/3 ms; flip angle 15°; image resolution 100 × 100 × 100 µm^[^ ^3^ ^]^; FOV 3 × 0.8 × 1.1 cm^2^; acquisition matrix 300 × 80 × 110) was used to acquire anatomical images. The contusion volumes were segmented manually by a trained expert using freely available ITK‐SNAP software.^[^ ^94^, ^95^ ^]^

### Blood Collection

The submandibular vein was accessed in order to acquire blood from awake mice and transferred immediately to a BD microvacutainer EDTA tube (Becton Dickinson CAT NUM). Blood samples were centrifuged at 5000 rcf for 5 min at 4 °C and the collected plasma was stored at −80 °C until analysis.

### Histology

Mice from cohort 2 were euthanized with deep anesthesia (ketamine 150 mg kg^−1^ and medetomidine 2 mg kg^−1^, i.p.) and transcardially perfused (30 mL of PBS 0.1 mol L^−1^, pH 7.4 followed by 60 mL of paraformaldehyde PFA 4% in PBS) as previously described.^[^ ^12^ ^]^ Serial coronal brain sections (20 µm) were cut on a cryostat (+1 to −4 mm from bregma) at 200 µm intervals for histological analysis and stained by Nissl or analyzed for pericontusional neuronal (anti‐NeuN 1:200; Millipore, #MAB377) and vessel (anti‐CD31, 1:100; BD bioscience, # 550 274) density as previously described.^[^ ^12^ ^]^

Mice from cohort 4 were euthanized as above and transcardially perfused (30 mL of PBS 0.1 mol L^−1^) and fixed in 10% formalin and embedded in paraffin as previously described.^[^ ^101^ ^]^ Serial sagittal sections (8 µm), ipsilateral to the injury, were cut, deparaffined (58 °C for 20 min), rehydrated, and processed for heat‐induced antigen retrieval (sodium citrate buffer pH 6.0 in a microwave for 5 min at 750 W). Microglial and astrocytic reactivity were examined by immunohistochemistry, by anti‐IBA1 (1:1000; Wako, #019‐19741) and anti‐Glial Fibrillary Acidic Protein (GFAP, 1:4000; Dako, #Z0334) as previously described.^[^ ^101^ ^]^

### Histological Quantification

Nissl‐stained sections were acquired at 2×, while immunostained sections (NeuN, CD31, IBA1, GFAP) at 20× magnification with an Olympus BX‐61‐VS microscope, interfaced with VS‐ASW‐FL software (Olympus). The acquisition was done over 10 µm (cohort 2) and 8 µm thick (cohort 4) stacks, with 2 µm step size. Images were analyzed using the Fiji software.

### Histological Quantification:Contusion and Hippocampal Volumes

Nissl‐stained coronal sections were used to evaluate contusion volume (Anteroposteriority, AP: +2 to −3.76 mm from bregma) or hippocampal volume (AP: −0.8 to −3.25 mm from bregma) as previously described.^[^ ^97^, ^98^ ^]^

### Histological Quantification:Neuronal and Vessel Density

Three NeuN or CD31 stained coronal sections (AP: −0.4, −1.6, −2.8 mm from bregma) were analyzed for each animal over a total area of 0.6 mm depth from the edge of the contusion. The degree of neuronal loss and vessel density were expressed as a percentage of the ipsilateral over contralateral hemisphere as previously described.^[^ ^12^, ^98^ ^]^

### Histological Quantification:Glial Activation

Two IBA1 or GFAP stained sagittal ipsilateral sections (Mediolaterality, ML: +1.1, +1.2 mm from midline) were analyzed for each animal. For each section, three adjacent ROIs (200 µm width) from the edge of the contusion were manually outlined on the cortex and corpus callosum. The immunostained area was expressed as percentage‐stained area.^[^ ^101^ ^]^

### RNA‐Seq and Analysis

RNA Sequencing (RNA‐Seq) was performed on the Illumina NextSeq500 with single‐end, 76 base pair long reads. The overall quality of sequencing reads was evaluated using FastQC (v.0.11.9).^[^ ^102^ ^]^ Sequence alignments of total‐RNA (stranded) to the reference mouse genome (GRCm39) were performed using STAR (v.2.7.9a)^[^ ^103^ ^]^ in two‐pass mode. Gene expression was quantified at the gene level by using the comprehensive annotations made available by Gencode (vM27 GTF File). Samples were adjusted for library size and normalized with the variance stabilizing transformation (vst) in the R statistical environment. Differential analysis was conducted with the DESeq2 (v1.28.1) pipeline.^[^ ^104^ ^]^ GSEA^[^ ^105^ ^]^ was performed using the Limma (v. 3.52.2)^[^ ^106^ ^]^ package and GSEABase (v 3.19). P‐values were corrected for multiple testing using the false discovery rate (FDR) procedure, with the significance threshold set to 0.05. The raw data are available in the Annotare database EMBL‐EBI^[^ ^107^ ^]^ (https://www.ebi.ac.uk/fg/annotare/) under the accession numbers: E‐MTAB‐14942.

### In Vitro TBI Model

Organotypic cortical brain slices were obtained under sterile conditions from the prefrontal cortex of C57BL/6J mouse pups (P1‐P3) and subjected to TBI by controlled cortical impact (Impact One, Leica) as previously described.^[^ ^13^ ^]^ One hour after TBI, culture medium was substituted with fresh NB/B27 (no treatment) or with NB/B27 containing SYNT.

At 48 h, slices were assessed for cell death by propidium iodide incorporation (PI) assay, then slices were collected for gene expression analysis as previously described.^[^ ^13^ ^]^ The primers used are listed in Supplementary Table S3 (Supporting Information).

Neuronal damage was assessed quantifying the levels of NfL released in culture medium using commercially available single molecule array assay kits (Quanterix, #103 400) on an SR‐X Analyzer (Quanterix), following manufacturer's instructions.^[^ ^13^, ^101^ ^]^

### Kynurenine Pathway Analysis

Medium from the in vitro TBI model was analyzed for the determination of Kynurenine pathway's metabolites kynurenic acid (KYNA) and 3‐hydroxyanthranilic acid (3HAA), broadly classified as neuroprotective and neurotoxic respectively.^[^ ^16^ ^]^ LC‐MRM analysis was performed on a triple‐quadrupole mass spectrometer (AB SCIEX triple‐quadrupole 5500) operating in MRM coupled with an Agilent 1260 Infinity II High‐Performance Liquid Chromatography (HPLC) system as previously reported.^[^ ^108^ ^]^ Twenty µL of culture media were deproteinized by mixing with four volumes of cold methanol, vortexed, and incubated at −80 °C for 20 min. Samples were then centrifuged 15 min at 14.000 × g, the supernatant was collected for the LC‐MS/MS analysis. The volume of injection was 8 µL.

### Circulating Proteins Analysis

Plasmatic samples were analyzed using the proximity extension assay (PEA) with the Target 96 Mouse Exploratory Panel (Olink Proteomics) as outlined previously.^[^ ^109^ ^]^ In this method, 92 specific target proteins bind to antibody probes labeled with double oligonucleotides. Following this, microfluidic real‐time PCR amplifies the oligonucleotide sequences to enable quantitative detection of DNA sequences. The threshold cycle (Ct) data derived from both internal and external controls underwent quality control and normalization processes. The protein levels were assessed on a relative scale and are presented as a normalized protein expression (NPX) unit, displayed on a log2 scale. A complete list of the proteins analyzed can be found in Supplementary Table S4 (Supporting Information).

### Plasma Biochemical Analysis

Plasma samples (100 µL) were analyzed through the Element Rc analyser (Antech Diagnostics Italy S.r.l., Treviglio, Italy) in order to quantify the amounts of total protein, Albumin, Globulin, Albumin/Globulin ratio, LDH, Glucose and Triglycerides levels to assess for the general state of health in mice, BUN, Creatinine, BUN/creatinine ratio, phosphorus, AST, total bilirubin and the creatine kinase to assess for kidney, liver and muscle function, respectively.

### Statistical Analysis

Data were shown as single values and/or mean ± standard error of the mean (SEM), or as single values and median + interquartile range with whiskers showing minimum and maximum values as indicated in the figure legends. Data were analyzed by: i) two‐way ANOVA for repeated measures followed by Tukey's post hoc test for longitudinal assessments (Neuroscore and SNAP tests, contusion volume assessed by (MRI), ii) unpaired t‐test (Y maze, contusion and hippocampal volumes assessed by Nissl staining, NeuN and CD31 histological staining), iii) one‐way ANOVA followed by Tukey's post hoc test (for all in vitro assessments) cell death, neuronal injury, gene expression), iv) two‐way ANOVA followed by Tukey's post hoc test (GFAP and IBA1 histological staining). *p‐*Values of 0.05 were considered statistically significant. Assumptions of normality were checked using the D'Agostino‐Pearson omnibus test. Outliers were identified using the ROUT method and excluded from the analyses. All the analyses above were performed with GraphPad Prism 9.0 Software.

Circulating protein levels by Olink Proteomics were analyzed by a linear random‐intercept regression model. Specifically, the NPX of each protein was regressed against the treatment group, the time, and the treatment‐time interaction. To account for the repeated measurements on mice, a mouse‐specific random intercept term was included. For each protein, F‐tests based on ad hoc contrasts were used to test whether the NPX differed among the treatment groups at 3 days or 1 month after injury. Inference was made on p‐values adjusted according to the Benjamini & Hochberg correction for multiple comparisons.^[^ ^110^ ^]^ Olink statistical analyses were performed with R, version 4.2.1,^[^ ^111^ ^]^ and used the package OlinkAnalyze, version 3.6.0,^[^ ^112^ ^]^ for data preprocessing.

All tests verified two‐sided alternative hypotheses and were considered as significant for *p* values <0.05.

## Conflict of Interest

The authors declare no conflict of interest.

## Author Contributions

F.P. and E.R.Z. conceived the study, designed experiments, and supervised the research; F.P., F.T., M.M., G.D.S., F.M., L.L., E.C., C.B., E.M., R.P., E.M., M.C.T., M.B., L.G., M.B.V., P.B., C.B., L.B., and E.R.Z. conducted the main experiments and associated analysis; G.N. and F.S. performed statistical analysis; F.P. and E.R.Z. wrote the manuscript with input from all authors; M.M., A.R.S., C.B., O.P., R.P., and L.B. revised the manuscript; F.P. and E.R.Z. acquired funding

## Supporting information



Supporting Information

Supplemental Table 1

Supplemental Table 2

Supplemental Table 3

Supplemental Table 4

## Data Availability

Raw data are available as supplementary data or at the following link: https://www.zenodo.org/records/15193778. RNAseq raw data are available at: https://www.ebi.ac.uk/fg/annotare/ under the accession numbers: E‐MTAB‐14942.
