# Supplementary material for: Mesenchymal Stromal Cell Secretome and Its Key Bioactive Metabolites Induce Long‐Term Neuroprotection After Traumatic Brain Injury in Mice
Source: Adv Sci (Weinh). 2025 Jun 19;12(29):e15508. doi: 10.1002/advs.202415508 (PMC12362754; doi:10.1002/advs.202415508)
Supplement: Supplementary file 1 — Supporting Information [file ADVS-12-e15508-s002.pdf]

ADVANCED SCIENCE
Open Access

Supporting Information

for *Adv. Sci.*, DOI 10.1002/advs.202415508

Mesenchymal Stromal Cell Secretome and Its Key Bioactive Metabolites Induce Long-Term Neuroprotection After Traumatic Brain Injury in Mice

Francesca Pischiutta, Francesca Tribuzio, Marta Magatti, Giulia De Simone, Federico Moro, Giovanni Nattino, Fabiola Signorini, Luther Loose, Enrico Caruso, Costanza Bertani, Edoardo Mazzone, Rosaria Pascente, Edoardo Micotti, Antonietta Rosa Silini, Fabrizio Ortolano, Maria Chiara Trolese, Marco Bolis, Luca Guarrera, Martina Bruna Violatto, Paolo Bigini, Cristina Banfi, Roberta Pastorelli, Ornella Parolini, Laura Brunelli and Elisa R Zanier**

Supplementary Figure 1

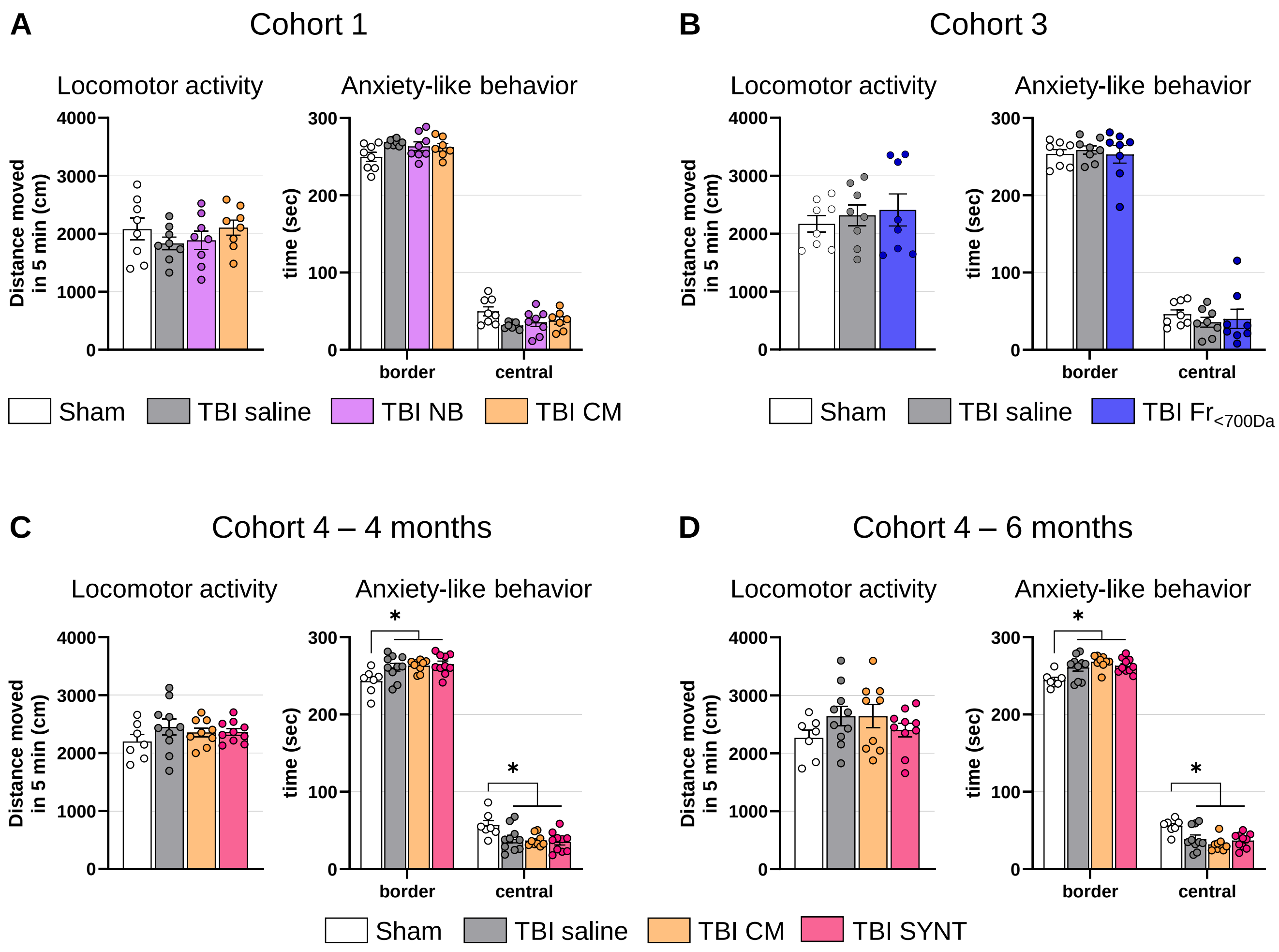

Supplementary Figure 1. **Open field test.** Assessment of locomotor activity (total trvelled distance) and anxiety-like behavior (time spent in center vs central zone), in Cohort 1 (A), Cohort 3 (B), both performed at 1 week, and in Cohort 4 at four (C) or six (D) months. In cohort 1 and 3, open field test performed at 1 week, revealed no significant difference between groups in terms of total distance travelled or time spent in border/central zone.

In cohort 4, at both 4 and 6 months, no significant difference were found in the total distance travelled. Compared to sham, TBI groups showed increased anxiety-like behavior revealed by higher time spent in the border zone and less in the center zone compared to sham. No treatment effects were observed. Data are presented as mean ± SEM; n=8-10. Total distance: One way ANOVA (ns); Center/border zone: Two-way ANOVA, followed by Tukey post-test. *p<0.05.

Supplementary Figure 2

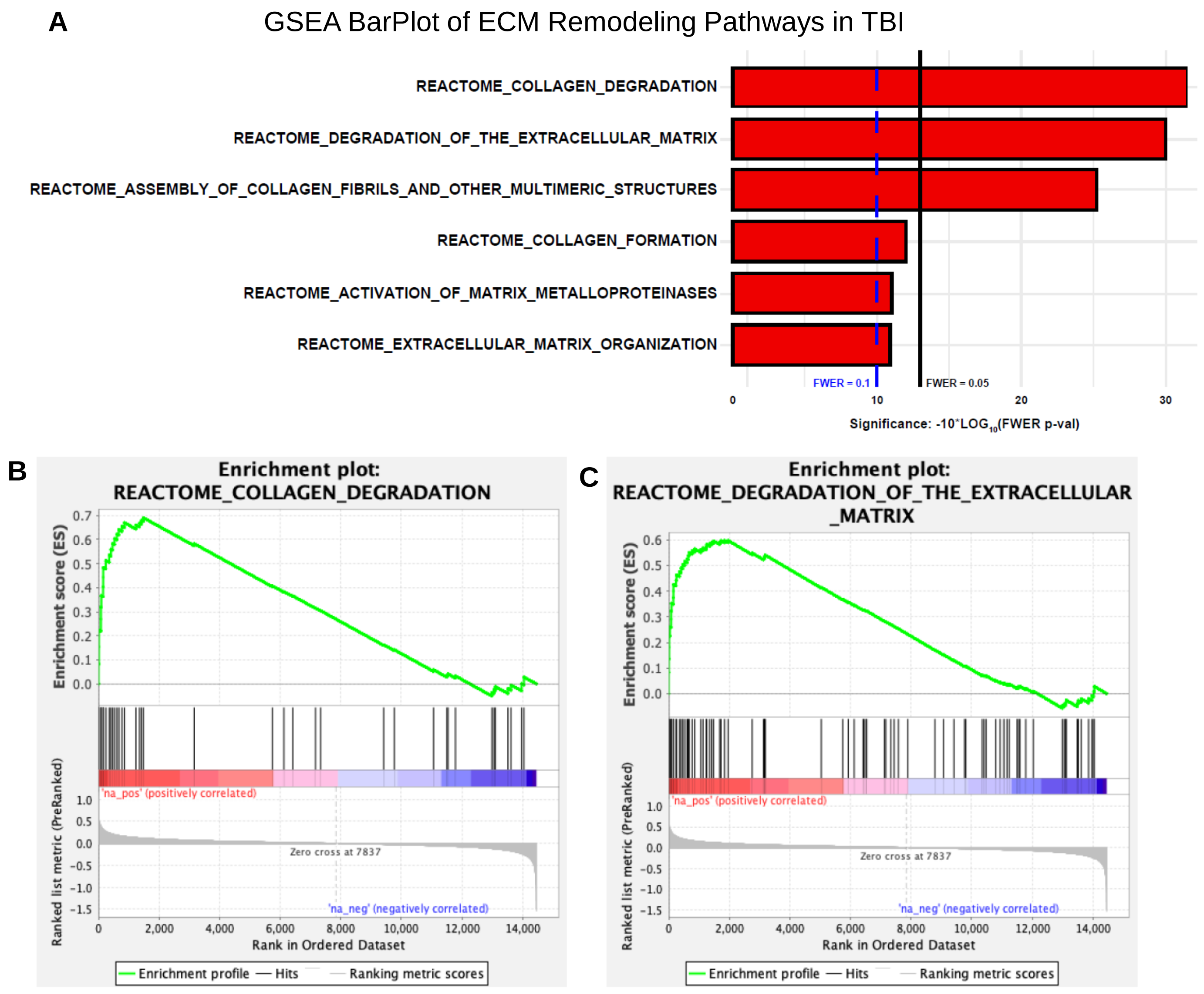

Supplementary Figure 2. **Gene Set Enrichment Analysis (GSEA) and Enrichment Map illustrating ECM remodeling after CM treatment in TBI mice. A)** Barplot illustrating the significance of extracellular matrix (ECM) remodeling pathways identified by Gene Set Enrichment Analysis (GSEA) in mice subjected to traumatic brain injury (TBI) and treated with conditioned medium (CM) compared to saline controls. Pathways are represented based on their Family-Wise Error Rate (FWER) p-value significance (-10 × log10 transformation). Red bars indicate pathways that are significantly upregulated following TBI. Dashed lines represent two significance thresholds: FWER = 0.1 and FWER = 0.05. The FWER p-value adjusts for multiple comparisons, controlling the probability of observing one or more false-positive results across all tested gene sets.

B) GSEA enrichment plot comparing TBI mice treated with CM versus saline controls for the "Reactome Collagen Degradation" pathway. The plot displays the running enrichment score (ES) in green, peaking at the highest enrichment point, indicating a positive correlation and significant upregulation of collagen degradation-related genes following CM treatment. Vertical black bars represent gene hits contributing most to the enrichment score.

C) GSEA enrichment plot comparing TBI mice treated with CM versus saline controls for the "Reactome Degradation of the Extracellular Matrix" pathway. The enrichment profile (ES) peaks early in the ranking, similarly indicating a positive correlation and significant enrichment of ECM degradation genes in the CM-treated group.

Supplementary Figure 3

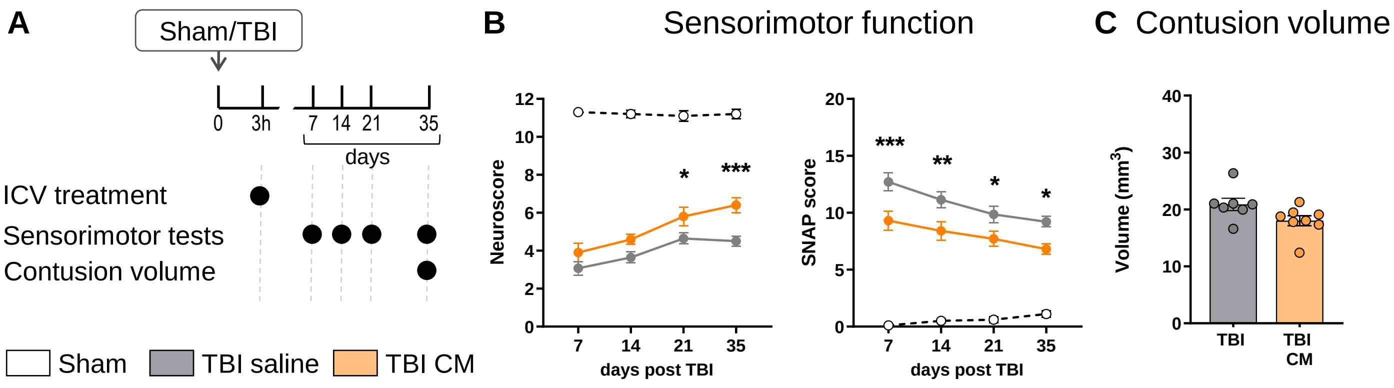

Supplementary Figure 3. **CM efficacy in TBI mice after intracerebroventricular infusion. A**) Schematic representation of the experimental design. **B**) Sensorimotor assessment up to 1-month post TBI by Neuroscore and SNAP tests showing significant improvement of sensorimotor function. Considering the area under the curve as comparator between ICV vs IP infusion, ICV induced an overall effect less pronounced than what observed with repeated IP administration: IP administration yielded improvements of 48% and 40% of SNAP and Neuroscore respectively, whereas single ICV administration resulted in 24% and 29% improvement. **C**) Quantification of contusion volume 1-month post-injury. Data are presented as mean ± SEM; n=7-8 mice/group. B) Two-way ANOVA for repeated measurement, followed by Tukey post test. C) t-test. *$p<0.05$, **$p<0.01$, ***$p<0.001$.

Supplementary Figure 4

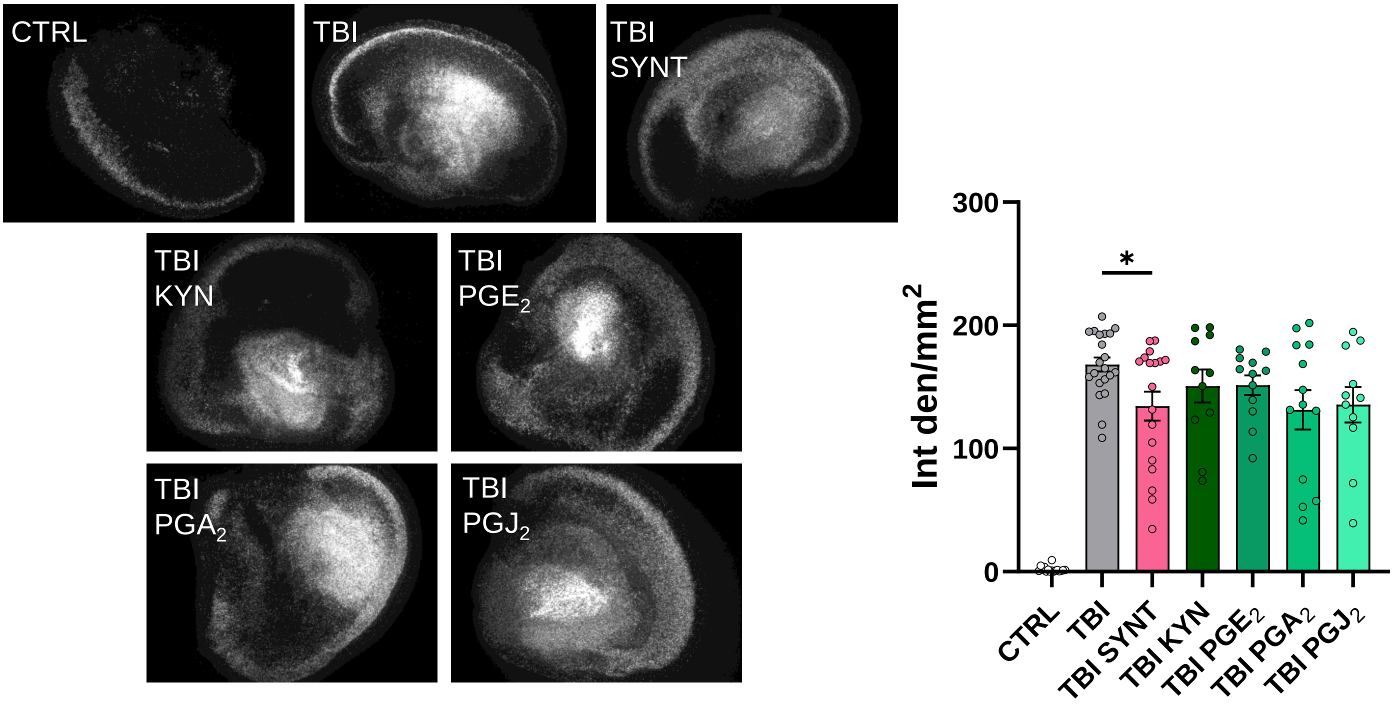

Supplementary Figure 4. **Efficacy of synthetic cocktail vs single factors after TBI in vitro.** Representative images showing propidium iodide (PI) incorporation 48 h after injury and their relative quantification. Data are presented as mean ± SEM from 2 independent experiments, n=6-8 each. One-way ANOVA, followed by Tukey post test. *p<0.05.

Supplementary Figure 5

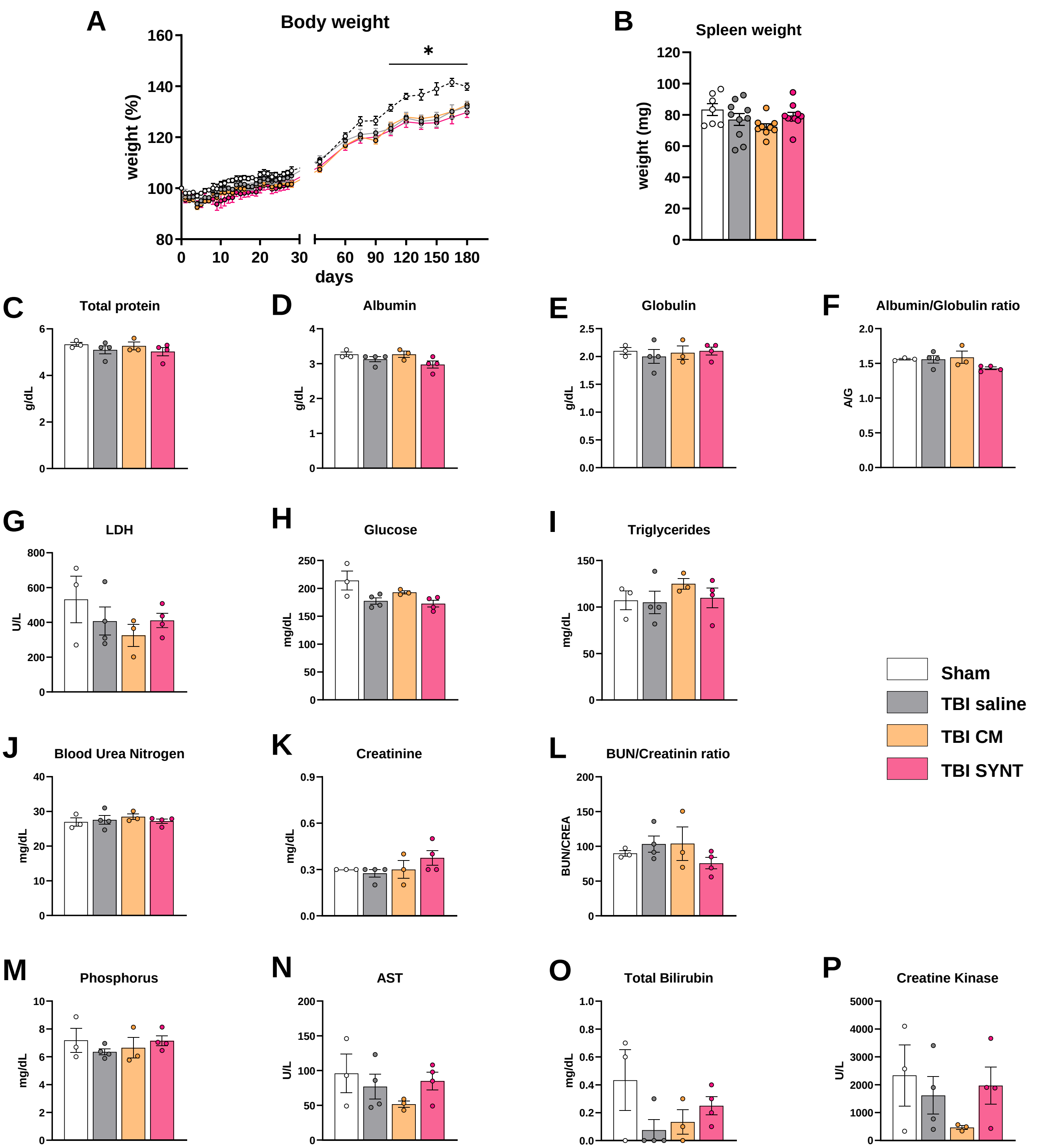

Supplementary Figure 5**. Assessment of possible toxic effect after repeated CM/SYNT infusion. A**) Longitudinal weight monitoring revealed a TBI effect on weight gain from 3.5 months on, however no significant treatment (CM nor SYNT) effects were recorded. **B**) No difference in the spleen weight was observed between the four experimental groups, excluding possible splenomegaly-associated toxic effects. **C-P**) The biochemical assessment of plasma samples revealed no difference in total protein (C), Albumin (D), Globulin (E), Albumin/Globulin ratio (F), LDH (G), Glucose (H), Triglycerides (I) levels, indicating that TBI nor treatments affect the general state of health in mice. In keeping with this, blood urea nitrogen (J), Creatinine (K), BUN/creatinine ratio (L), phosphorus (M), AST (N), total bilirubin (O) and the creatine kinase (P) levels were unchanged between the four experimental groups, indicating the correct maintenance of kidney, liver and muscle function, respectively. Data are presented as mean ± SEM; n=8-10 (A-B), n=3-4 (C-P), by RM-ANOVA (A) or one-way ANOVA (B-P), followed by Tukey post hoc test. *$p<0.05$.
