# Supplementary material for: Mesenchymal Stromal Cell Secretome and Its Key Bioactive Metabolites Induce Long‐Term Neuroprotection After Traumatic Brain Injury in Mice
Source: Adv Sci (Weinh). 2025 Jun 19;12(29):e15508. doi: 10.1002/advs.202415508 (PMC12362754; doi:10.1002/advs.202415508)
Supplement: Supplementary file 2 — Supplemental Table 1 [file ADVS-12-e15508-s005.docx]

**Supplemental Table 1**: Transitions of the selected metabolites identified during the optimization of the method

| **Classes** | **Name** | **Polarity** | **Precursor Ion (m/Z)** | **Target Ion (m/Z)** | **Confirmation Ion (m/Z)** |
| --- | --- | --- | --- | --- | --- |
|  |  |  |  |  |  |
| **Nucleic acid related** | Cytidine monophosphate | + | 324.0 | 112.5 | 95.1 |
|  | Guanosine monophosphate | + | 364.0 | 152.3 | 135.1 |
|  | Uracil | + | 113.0 | 70.3 | 96.15 |
|  | Adenosine monophosphate | + | 348 | 136.2 | 119.20 |
|  | Hypoxanthine | + | 137.0 | 119.2 | 110.2 |
|  | Uridine | + | 245.0 | 113.3 | 96.15 |
|  | Thymine | + | 127.1 | 110.05 | 54.3 |
|  | Inosine | + | 269.1 | 137.2 | 110.15 |
|  | Guanosine | + | 284 | 152.1 | 135.2 |
|  | Xanthosine | + | 284.9 | 153.25 | 136.25 |
|  | Cytidine | + | 244.1 | 112.3 | 95.25 |
|  | Thymidine | + | 243.1 | 127.3 | 110.3 |
|  | Adenine | + | 136.0 | 119.2 | 92.2 |
|  | Adenosine | + | 268.1 | 136.05 | 119.1 |
|  | Deoxycytidine | + | 228.1 | 112.05 | 95.15 |
|  | Uric Acid | - | 167.10 | 124.15 | 96.00 |
|  | Xanthine | - | 151.00 | 108.20 | 66.20 |
|  | Guanine | - | 150.00 | 133.20 | 66.05 |
|  | Niacinamide | + | 123.00 | 80.10 | 78.10 |
| **Sugar** | Glucosamine | + | 180.0 | 162,25 | 72,25 |
|  | Gluconic acid | - | 195.2 | 75 | 129.2 |
|  | Threonic acid | - | 135.2 | 74.95 | 58.95 |
|  | Sucrose | - | 341.3 | 89.2 | 179.1 |
| **Vitamin** | Nicotinic Acid | + | 124.05 | 80.1 | 78.0 |
|  | Choline | + | 104.1 | 60.5 | 58.25 |
|  | Pantothenic Acid | + | 220.1 | 90.15 | 202.1 |
|  | Pyridoxal | + | 167.9 | 150.0 | 94.15 |
|  | 4-Aminobenzoic Acid | + | 138.25 | 65.1 | 77.0 |
|  | Riboflavin | + | 377 | 243.1 | 172.25 |
|  | Biotin | + | 245.1 | 97.1 | 123.1 |
|  | Ergocalciferol | + | 397.35 | 69.5 | 105.05 |
|  | Ascorbic Acid | - | 175.2 | 86.85 | 130.8 |
|  | Cyanocobalamine | - | 1354.5 | 339.00 | 132 |
|  | Pyridoxine | - | 169.9 | 152.00 |  |
|  | Lipoic Acid | - | 205.2 | 171.2 | 127.3 |
|  | Tocopherol acetate | + | 473.0 | 207.15 | 165.1 |
| **Amino Acid related** | Cystine | + | 241.0 | 151.95 | 120.05 |
|  | Asparagine | + | 133.1 | 87.2 | 28.1 |
|  | Aspartic Acid | + | 134 | 74.05 | 88.2 |
|  | Serine | + | 105.9 | 60.1 | 42.15 |
|  | 4-Hydroxyproline | + | 132.1 | 86.1 | 68.1 |
|  | Glycine | + | 75.9 | 30.0 |  |
|  | Glutamine | + | 145.1 | 127.2 | 190.1 |
|  | Cysteine | + | 122.0 | 76.1 | 59.1 |
|  | Threonine | + | 120.1 | 74.05 | 56.1 |
|  | Methionine Sulfoxide | + | 166.0 | 73.95 | 56.2 |
|  | Glutamic Acid | + | 147.9 | 84.15 | 56.1 |
|  | Alanine | + | 79.9 | 44.15 |  |
|  | Citrulline | + | 176.1 | 70.1 | 159.05 |
|  | Ornithine | + | 133.1 | 70.1 | 116.05 |
|  | Proline | + | 116.1 | 70.2 | 28.15 |
|  | Lysine | + | 147.2 | 84.1 | 130.1 |
|  | Histidine | + | 155.9 | 110.1 | 83.1 |
|  | Arginine | + | 175.2 | 70.1 | 60.1 |
|  | N-Acetylaspartic Acid | + | 175.9 | 88.25 | 74.1 |
|  | 4-Aminobutyric Acid | + | 104.0 | 87.2 | 69.1 |
|  | Glutathione | + | 308.0 | 178.7 | 162 |
|  | Valine | + | 118.0 | 72.1 | 55.15 |
|  | Methionine | + | 149.9 | 56.2 | 61.05 |
|  | N-Acetylcysteine | + | 164.1 | 122.1 | 75.95 |
|  | Tyrosine | + | 182.1 | 91.1 | 136 |
|  | Isoleucine | + | 132.1 | 86.1 | 69.1 |
|  | Leucine | + | 132.1 | 86.15 | 30.05 |
|  | Phenylalanine | + | 166.1 | 120.1 | 103.05 |
|  | Kynurenine | + | 209.1 | 192.1 | 146.05 |
|  | Tryptophan | + | 205.1 | 188.1 | 118.1 |
|  | Cystathione | + | 223 | 88.05 | 134 |
|  | Amino adipic acid | + | 162.2 | 55.05 | 116.3 |
|  | 5-oxoproline | - | 128.2 | 84 | 82.2 |
| **Glycolysis-TCA cycle related** | Hexose (Glucose) | - | 179.2 | 88.93 |  |
|  | Malate | - | 133.1 | 115.5 | 71.15 |
|  | Pyruvate | - | 86.90 | 43.0 |  |
|  | Lactate | - | 89.30 | 43.15 |  |
|  | Citrate | - | 191.2 | 111.2 | 85.0 |
|  | Succinate | - | 117.3 | 73.05 |  |
|  | Fumarate | - | 115.0 | 71.1 | 27.15 |
| **Other** | Glyceric Acid | - | 105.2 | 75.1 | 56.9 |
|  | 2-Aminoethanol | + | 62.15 | 45.1 |  |
|  | Ethylenediamine | + | 61.15 | 44.25 |  |
|  | Putrescine | + | 89.2 | 72.35 | 30.2 |
|  | O-Phosphoethanolamine | + | 142.1 | 44.1 |  |
|  | Histamine | + | 112.1 | 95.2 | 41.25 |
