# Supplementary material for: Mesenchymal Stromal Cell Secretome and Its Key Bioactive Metabolites Induce Long‐Term Neuroprotection After Traumatic Brain Injury in Mice
Source: Adv Sci (Weinh). 2025 Jun 19;12(29):e15508. doi: 10.1002/advs.202415508 (PMC12362754; doi:10.1002/advs.202415508)
Supplement: Supplementary file 3 — Supplemental Table 2 [file ADVS-12-e15508-s004.docx]

**Supplemental Table 2**: transitions of the selected metabolites identified during the optimization of the method

| **Classes** | **Name** | **Polarity** | **Precursor Ion (m/Z)** | **Target Ion (m/Z)** | **Confirmation Ion (m/Z)** |
| --- | --- | --- | --- | --- | --- |
|  |  |  |  |  |  |
| **AA related pro-inflammatory** | AA |  | 303.3 | 259.3 | - |
|  | PGD2 | - | 351.2 | 315.2 | 271.25 |
|  | PGE2 | - | 351.2 | 315.2 | 333.15 |
|  | PGF2alpha | - | 353.2 | 193.15 | 291.15 |
|  | PGJ2 | - | 333.2 | 121 | 241 |
|  | PGA2 | - | 333.2 | 207.1 | 161.3 |
|  | 6-keto-PGF1aplha | - | 369.4 | 245.15 | 163.15 |
|  | 15-deoxy-PGJ2 | - | 315.2 | 271.15 | 203.15 |
|  | TXB2 | - | 369.4 | 169.2 | 195.05 |
|  | LTB4 | - | 335.4 | 195.3 | 317.25 |
|  | LTC4 | - | 624.3 | 272.1 | 254.1 |
|  | 12-(S)-HETE | - | 319.2 | 179.3 | 301.45 |
|  | 14,15-EET | - | 319.4 | 301.25 | 109 |
|  | 5-(S)-HETE | - | 319.2 | 301.15 | 115.1 |
|  | 14-(15)-DiHET | - | 337.4 | 207.2 | 129.15 |
|  | AA-d11 | - | 314.7 | 270.25 | - |
|  | PGE2-d4 | - | 355.2 | 275.2 | 337.25 |
|  | LTB4-d4 | - | 339.4 | 197.15 | 321.2 |
|  | 5-(S)-HETE-d8 | - | 327.2 | 309.15 | 116.2 |
| **AA related pro-resolving** | 15-(S)-HETE | - | 319.2 | 301.1 | 219.2 |
|  | LXA4 | - | 351.4 | 217.4 | 235.1 |
|  | LXB4 | - | 351.2 | 221.2 | 233 |
|  | LXA4-d5 | - | 356.4 | 222.2 | 240.15 |
| **EPA related pro-resolvin** | EPA | - | 301.5 | 257.2 | - |
|  | 18-HETE | - |  |  |  |
|  | RE1 | - | 349.2 | 195.2 | 161.3 |
|  | EPA-d5 | - | 306.7 | 262.25 | - |
|  | RE1-d4 | - | 353.5 | 197.1 | 109.3 |
| **DHA releted pro-resolving** | DHA | - | 327.5 | 283.1 | - |
|  | RD1 | - | 375.2 | 141.2 | 121.15 |
|  | RD2 | - | 375.2 | 175.15 | 329.15 |
|  | RD3 | - | 375.2 | 147.3 | 95.2 |
|  | RD4 | - | 375.2 | 101 | 225.25 |
|  | RD5 | - | 359.2 | 199.1 | 297 |
|  | PDX | - | 359.2 | 153.25 | 206.15 |
|  | Maresin1 | - | 359.4 | 177.3 | 250.1 |
|  | Maresin2 | - | 359.4 | 221.25 | 232.1 |
|  | RD2-d5 | - | 380.5 | 277.15 | 141.25 |
|  | Maresin1-d5 | - | 364.4 | 93.15 | 250.4 |
| **Linolenic acid related pro-inflammatory** | 9-(S)-HODE | - | 295.3 | 277.15 | 171.3 |
|  | 9-(10)-DiHOME | - | 313.3 | 201.3 | 171.3 |
|  | 12-(13)-DiHOME | - | 313.3 | 183.3 | 129.3 |
| **Linolenic acid related pro-resolving** | 13-(S)-HODE | - | 295.3 | 277.2 | 195.1 |
| **AEA related pro-resolvin** | Arachidonoyl-ethanolamide (AEA) | + | 348 | 62.1 | 287.2 |
|  | PGE2-EA | + | 396 | 378.05 | 360.35 |
|  | PGF2-EA | + | 397 | 328.75 | 351 |
|  | 5,6-EET EA | + | 364 | 346.3 | 98.1 |
