# Supplementary material for: Mesenchymal Stromal Cell Secretome and Its Key Bioactive Metabolites Induce Long‐Term Neuroprotection After Traumatic Brain Injury in Mice
Source: Adv Sci (Weinh). 2025 Jun 19;12(29):e15508. doi: 10.1002/advs.202415508 (PMC12362754; doi:10.1002/advs.202415508)
Supplement: Supplementary file 4 — Supplemental Table 3 [file ADVS-12-e15508-s001.docx]

Supplementary Table 3 – Gene primer sequences for RT-PCR analyses

| **Gene** | **Primer *Forward*** | **Primer *Reverse*** |
| --- | --- | --- |
| ***RPL27*** | TCATGAAACCCGGGAAAGT | GAGGTGCCATCGTCAATGT |
| ***NeuN*** | CAGACGGTGCCGCAGG | ATGTAGTCGTTTGGGCTGCT |
| ***BDNF*** | AGGCACTGGAACTCGCAATG | AAGGGCCCGAACATACGATT |
| ***IL-6*** | GCTTAATTACACATGTTCTCTGGGAAA | CAAGTGCATCATCGTTGTTCATAC |
| ***CD11b*** | GAGCAGCACTGAGATCCTGTTTAA | ATACGACTCCTGCCCTGGAA |
| ***CD86*** | GTTACTGTGGCCCTCCTCCTT | CTGATTCGGCTTCTTGTGACATA |
| ***Arg1*** | CATGGGCAACCTGTGTCCTT | TCCTGGTACATCTGGGAACTTTC |
| ***CD206*** | CCCAAGGGCTCTTCTAAAGCA | CGCCGGCACCTATCACA |
| ***GFAP*** | GAAAACCGCATCACCATTCC | TCGGATCTGGAGGTTGGAGA |
| ***Serping1*** | ACAGCC CCCTCTGAATTCTT | GGATGCTCTCCAAGTTGCTC |
| ***Ggta1*** | GTTTTGTTGCCTCTGGGTGT | GTGAACAGCATGAGGGGTTT |
| ***H2-D1*** | TCCGAGATT GTAAAGCGTGAAGA | ACAGGGCAGTGCAGGGATAG |
| ***H2-T23*** | GGACCG CGAATGACATAGC | GCACCTCAGGGTGACTTCAT |
| ***Tgm1*** | CTGTTGGTCCCGTCCCAAA | GGACCTTCCATTGTGCCTGG |
| ***Clcf1*** | CTTCAATCCTCCTCGACTGG | TACGTCGGAGTTCAGCTGTG |
| ***S100a10*** | CCTCTGGCTGTGGACAAAAT | CTGCTCACAAGAAGCAGTGG |
