# Supplementary material for: Mesenchymal Stromal Cell Secretome and Its Key Bioactive Metabolites Induce Long‐Term Neuroprotection After Traumatic Brain Injury in Mice
Source: Adv Sci (Weinh). 2025 Jun 19;12(29):e15508. doi: 10.1002/advs.202415508 (PMC12362754; doi:10.1002/advs.202415508)
Supplement: Supplementary file 5 — Supplemental Table 4 [file ADVS-12-e15508-s003.docx]

Supplementary Table 4 – Target proteins of Olink Target 96 Mouse Exploratory plate

| **UniProt ID** | **Gene** | **Protein name** |
| --- | --- | --- |
| **P20826** | **Kitlg** | Kit ligand |
| **Q9D6N1** | **Ca13** | Carbonic anhydrase 13 |
| **P47713** | **Pla2g4a** | Cytosolic phospholipase A2 |
| **P10855** | **Ccl3** | C-C motif chemokine 3 |
| **P48787** | **Tnni3** | Troponin I, cardiac muscle |
| **Q04736** | **Yes1** | Tyrosine-protein kinase Yes |
| **Q00493** | **Cpe** | Carboxypeptidase E |
| **P12960** | **Cntn1** | Contactin-1 |
| **Q9JLL0** | **Crim1** | Cysteine-rich motor neuron 1 protein |
| **Q8R5A3** | **Apbb1ip** | Amyloid beta A4 precursor protein-binding family B member 1-interacting protein |
| **Q61865** | **Mia** | Melanoma-derived growth regulatory protein |
| **P25446** | **Fas** | Tumor necrosis factor receptor superfamily member 6 |
| **Q8VCF1** | **Cant1** | Soluble calcium-activated nucleotidase 1 |
| **Q99JW5** | **Epcam** | Epithelial cell adhesion molecule |
| **Q8CD15** | **Riox2** | Ribosomal oxygenase 2 |
| **P48540** | **Gdnf** | Glial cell line-derived neurotrophic factor |
| **Q6PCX7** | **Rgma** | Repulsive guidance molecule A |
| **P31240** | **Pdgfb** | Platelet-derived growth factor subunit B |
| **P08505** | **Il6** | Interleukin-6 |
| **P47931** | **Fst** | Follistatin |
| **O35625** | **Axin1** | Axin-1 |
| **Q62386** | **Il17a** | Interleukin-17A |
| **P07321** | **Epo** | Erythropoietin |
| **P58058** | **Nadk** | NAD kinase |
| **Q9R1E0** | **Foxo1** | Forkhead box protein O1 |
| **Q80UG2** | **Plxna4** | Plexin-A4 |
| **Q8CGN5** | **Plin1** | Perilipin-1 |
| **Q7TQN3** | **Wfikkn2** | WAP, Kazal, immunoglobulin, Kunitz and NTR domain-containing protein 2 |
| **P10148** | **Ccl2** | C-C motif chemokine 2 |
| **P30561** | **Ahr** | Aryl hydrocarbon receptor |
| **P18406** | **Ccn1** | CCN family member 1 |
| **Q8BLU0** | **Flrt2** | Leucine-rich repeat transmembrane protein FLRT2 |
| **Q9Z0T9** | **Itgb6** | Integrin beta-6 |
| **Q9R1V7** | **Adam23** | Disintegrin and metalloproteinase domain-containing protein 23 |
| **P97785** | **Gfra1** | GDNF family receptor alpha-1 |
| **Q9DCL8** | **Ppp1r2** | Protein phosphatase inhibitor 2 |
| **Q8BVI4** | **Qdpr** | Dihydropteridine reductase |
| **Q09163** | **Dlk1** | Protein delta homolog 1 |
| **P17183** | **Eno2** | Gamma-enolase |
| **Q8R373** | **Clmp** | CXADR-like membrane protein |
| **P30882** | **Ccl5** | C-C motif chemokine 5 |
| **O08712** | **Tnfrsf11b** | Tumor necrosis factor receptor superfamily member 11B |
| **P04202** | **Tgfb1** | Transforming growth factor beta-1 proprotein |
| **P48030** | **Tgfa** | Protransforming growth factor alpha |
| **P99029** | **Prdx5** | Peroxiredoxin-5, mitochondrial |
| **O08746** | **Matn2** | Matrilin-2 |
| **Q61982** | **Notch3** | Neurogenic locus notch homolog protein 3 |
| **Q61527** | **Erbb4** | Receptor tyrosine-protein kinase erbB-4 |
| **P55095** | **Gcg** | Pro-glucagon |
| **Q9ER65** | **Clstn2** | Calsyntenin-2 |
| **P07091** | **S100a4** | Protein S100-A4 |
| **Q9ERB0** | **Snap29** | Synaptosomal-associated protein 29 |
| **P01582** | **Il1a** | Interleukin-1 alpha |
| **Q4V9Z5** | **Sez6l2** | Seizure 6-like protein 2 |
| **Q8K4B4** | **Il23r** | Interleukin-23 receptor |
| **Q61483** | **Dll1** | Delta-like protein 1 |
| **Q9CWS0** | **Ddah1** | N(G),N(G)-dimethylarginine dimethylaminohydrolase 1 |
| **Q08048** | **Hgf** | Hepatocyte growth factor |
| **Q8BTW9** | **Pak4** | Serine/threonine-protein kinase PAK 4 |
| **Q9CR75** | **Tnfrsf12a** | Tumor necrosis factor receptor superfamily member 12A |
| **P12850** | **Cxcl1** | Growth-regulated alpha protein |
| **P18893** | **Il10** | Interleukin-10 |
| **P97326** | **Cdh6** | Cadherin-6 |
| **Q61288** | **Acvrl1** | Serine/threonine-protein kinase receptor R3 |
| **O89017** | **Lgmn** | Legumain |
| **O88393** | **Tgfbr3** | Transforming growth factor beta receptor type 3 |
| **Q6ZQA6** | **Igsf3** | Immunoglobulin superfamily member 3 |
| **P01587** | **Csf2** | Granulocyte-macrophage colony-stimulating factor |
| **P70236** | **Map2k6** | Dual specificity mitogen-activated protein kinase kinase 6 |
| **Q9R000** | **Itgb1bp2** | Integrin beta-1-binding protein 2 |
| **Q7TNI7** | **Il17f** | Interleukin-17F |
| **P10749** | **Il1b** | Interleukin-1 beta |
| **P70677** | **Casp3** | Caspase-3 |
| **O54775** | **Ccn4** | CCN family member 4 |
| **P18340** | **Cxcl9** | C-X-C motif chemokine 9 |
| **Q9Z109** | **Vsig2** | V-set and immunoglobulin domain-containing protein 2 |
| **P04401** | **Il5** | Interleukin-5 |
| **P26323** | **Fli1** | Friend leukemia integration 1 transcription factor |
| **O89093** | **Ccl20** | C-C motif chemokine 20 |
| **Q8BYI9** | **Tnr** | Tenascin-R |
| **O54907** | **Tnfsf12** | Tumor necrosis factor ligand superfamily member 12 |
| **P20181** | **Ntf3** | Neurotrophin-3 |
| **P97946** | **Vegfd** | Vascular endothelial growth factor D |
| **Q8BX35** | **Eda2r** | Tumor necrosis factor receptor superfamily member 27 |
| **Q99KJ8** | **Dctn2** | Dynactin subunit 2 |
| **P11103** | **Parp1** | Poly [ADP-ribose] polymerase 1 |
| **Q9EQC7** | **Fstl3** | Follistatin-related protein 3 |
| **P11152** | **Lpl** | Lipoprotein lipase |
| **P06804** | **Tnf** | Tumor necrosis factor |
| **O89023** | **Tpp1** | Tripeptidyl-peptidase 1 |
| **Q69Z26** | **Cntn4** | Contactin-4 |
| **Q9EQX0** | **Ghrl** | Appetite-regulating hormone |
